# Polychaete Richness and Abundance Enhanced in Anthropogenically Modified Estuaries Despite High Concentrations of Toxic Contaminants

**DOI:** 10.1371/journal.pone.0077018

**Published:** 2013-09-30

**Authors:** Katherine A. Dafforn, Brendan P. Kelaher, Stuart L. Simpson, Melinda A. Coleman, Pat A. Hutchings, Graeme F. Clark, Nathan A. Knott, Martina A. Doblin, Emma L. Johnston

**Affiliations:** 1 Evolution and Ecology Research Centre, School of Biological, Earth and Environmental Sciences, University of New South Wales, Sydney, NSW, Australia; 2 Sydney Institute of Marine Science, Mosman, NSW, Australia; 3 National Marine Science Centre, Southern Cross University, Coffs Harbour, NSW, Australia; 4 Commonwealth Scientific and Industrial Research Organisation, Land and Water, Kirrawee, NSW, Australia; 5 Aquatic Ecosystem Research, New South Wales Department of Primary Industries, Nelson Bay, NSW, Australia; 6 Marine Invertebrates, Australian Museum, Sydney, NSW, Australia; 7 University of Technology Sydney, Broadway, NSW, Australia; University of Southampton, United Kingdom

## Abstract

Ecological communities are increasingly exposed to multiple chemical and physical stressors, but distinguishing anthropogenic impacts from other environmental drivers remains challenging. Rarely are multiple stressors investigated in replicated studies over large spatial scales (>1000 kms) or supported with manipulations that are necessary to interpret ecological patterns. We measured the composition of sediment infaunal communities in relation to anthropogenic and natural stressors at multiple sites within seven estuaries. We observed increases in the richness and abundance of polychaete worms in heavily modified estuaries with severe metal contamination, but no changes in the diversity or abundance of other taxa. Estuaries in which toxic contaminants were elevated also showed evidence of organic enrichment. We hypothesised that the observed response of polychaetes was not a ‘positive’ response to toxic contamination or a reduction in biotic competition, but due to high levels of nutrients in heavily modified estuaries driving productivity in the water column and enriching the sediment over large spatial scales. We deployed defaunated field-collected sediments from the surveyed estuaries in a small scale experiment, but observed no effects of sediment characteristics (toxic or enriching). Furthermore, invertebrate recruitment instead reflected the low diversity and abundance observed during field surveys of this relatively ‘pristine’ estuary. This suggests that differences observed in the survey are not a direct consequence of sediment characteristics (even severe metal contamination) but are related to parameters that covary with estuary modification such as enhanced productivity from nutrient inputs and the diversity of the local species pool. This has implications for the interpretation of diversity measures in large-scale monitoring studies in which the observed patterns may be strongly influenced by many factors that covary with anthropogenic modification.

## Introduction

The intense and extensive development by humans across the planet has subjected much of the world's biological diversity to multiple severe anthropogenic stressors [1], [2], [3] including frequent chemical and physical disturbances, which are often concentrated in urban and industrial areas [4]. Human activity is often concentrated around waterways and associated activities inevitably release contaminants into water bodies and result in other modifications to physico-chemical conditions. As a consequence estuaries in particular have been highly impacted by stressors related to agriculture, industrialisation and urbanisation with almost all estuaries suffering some degree of impact [5]. Apart from physical modification to these systems (e.g. addition of artificial structures), important chemical stressors include toxic contaminants (e.g. metals and hydrophobic organic chemicals such as polycyclic aromatic hydrocarbons (PAHs)) [6], enriching contaminants (e.g. nutrients) [7], and changes to natural conditions such as temperature and salinity that are outside the range of natural variation [8], [9]. While much is understood of the effects of individual contaminants [10] and there have been limited comparisons of multiple stressors [7], [11], [12], [13], little is known of whether these effects will extend over large-spatial scales and if the ecology of these systems may influence these effects (e.g. the diversity and resilience of the assemblages, and potential for adaptation over long periods of time).

Environmental monitoring studies often rely on ecological measures of community composition and diversity as indicators of anthropogenic change [14], [15]. Such measures are strongly influenced by regional conditions and patterns may not always reflect impacts [16], [17]. Recent efforts to monitor ecological impacts have focused on integrating information collected from chemical and ecological monitoring into a more holistic understanding of ecosystem condition [18], [19], [20]. How this information is collected remains fiercely debated and ranges from laboratory-based experiments, field-based manipulative experiments (generally small-scale), and small- and large-scale observational studies (either snapshot or examining change through time). These each have advantages and disadvantages [21], [22], but to distinguish the effects of anthropogenic stressors from other environmental drivers [23] and inform management-oriented biomonitoring requires multiple lines of evidence and experimental studies in order to reliably interpret patterns of community composition and diversity [22], [24], [25].

Chemical stressors that are released into estuaries accumulate in benthic sediments [13], [26] and impact the ecological composition and function of this important habitat [27]. When tested individually and under laboratory conditions, contaminants such as metals and PAHs are known to have toxic effects on aquatic taxa including increased mortality [7], [11], [12], reduced reproductive potential [13], [28] and other sublethal effects [29]. Similarly, field surveys suggest that metal and organic chemical contamination are related to changes to community composition that can include the loss of sensitive species and the increased dominance of more tolerant taxa or individuals. A meta-analysis by [30] determined species richness was reduced by ∼40% across contaminated marine communities and the majority of the studies reviewed were conducted in soft sediment communities using field surveys. Contaminants such as dissolved nitrogen and phosphorus also have community-wide effects, but high levels can result in a community composed of very high densities of a few tolerant opportunistic species [31]. In contrast to toxic contaminants, however, such nutrients initially have an enriching effect with the result being observed increases in richness and abundance of primary producers and this has consequences at higher trophic levels [32].

We tested whether impacts of large-scale anthropogenic stress in estuaries could be detected over and above natural spatial variation across regional scales (100 s of kms) using an observational approach to assessing the outcome of contamination on benthic diversity. We surveyed benthic sediments in three heavily modified and four relatively ‘pristine’ estuaries [33] and we sampled infaunal assemblages, measures of toxic contaminants (metal and PAH concentrations) and indicators of organic enrichment (sediment chlorophyll-a levels, porewater ammonia and total organic carbon) and sediment grain size. We also measured other environmental variables (e.g. temperature and salinity). To aid our understanding of why differences in surveyed infaunal community composition (dominated by polychaetes) occurred in relation to anthropogenic modification, we undertook manipulative field experiments. We redeployed sediments collected from the estuaries with varying degrees of anthropogenic impacts into a single clean estuary and assessed the community composition and diversity of recruiting fauna.

## Materials and Methods

### Ethics Statement

This study surveyed benthic invertebrate distributions across estuaries and experiments were conducted on invertebrate communities. Sampling in each location was approved and carried out in strict accordance with the New South Wales Department of Primary Industries and New South Wales Marine Parks (Permit No. P09/0072-1.0).

### Field surveys of benthic sediments in multiple estuaries

**Figure 1:**
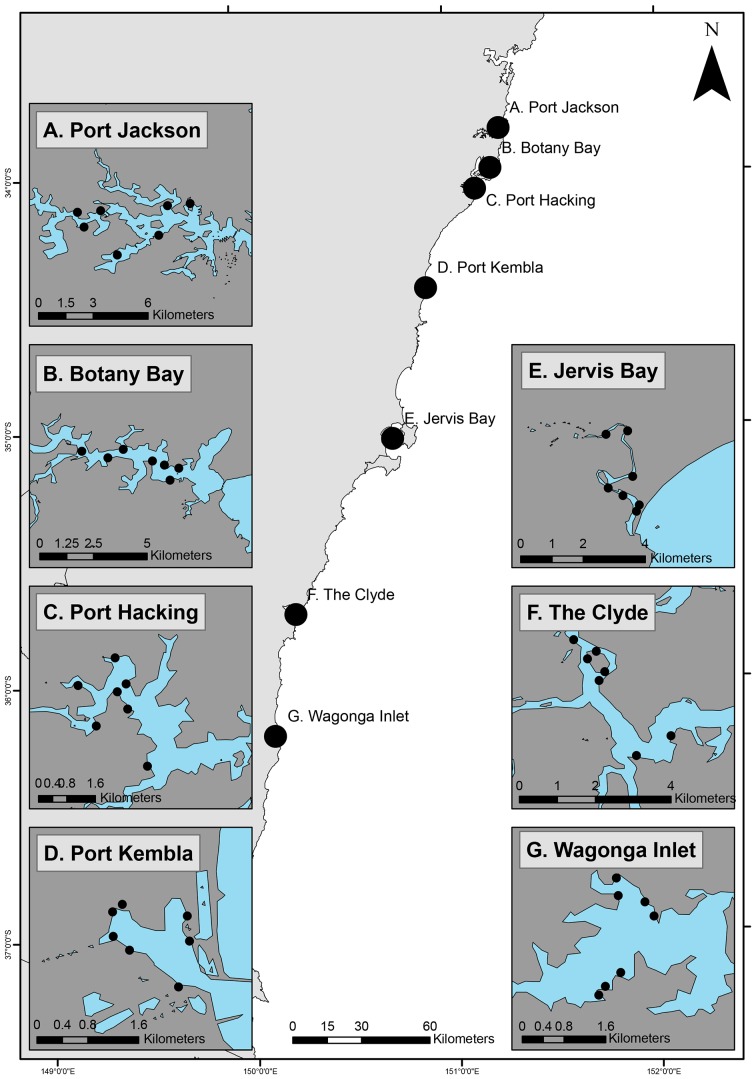
Locations of study sites along the coastline of New South Wales, south-east Australia. A. Port Jackson, B. Botany Bay and D. Port Kembla are heavily modified estuaries. C. Port Hacking, E. Jervis Bay, F. The Clyde and G. Wagonga Inlet are relatively unmodified estuaries.

We investigated variation in soft-sediment assemblages in relation to anthropogenic contaminants and other environmental variables at seven sites within seven estuaries. Estuaries were assigned a ‘modification category’ according to the level of anthropogenic modification and the study was conducted in three heavily modified and four relatively unmodified estuaries stretching across 400 km of the coast of New South Wales, Australia (Figure 1). In this study, heavily modified estuaries represented systems both physically modified and contaminant impacted by anthropogenic activities while relatively unmodified estuaries were systems lacking extensive urbanisation or industry. Port Kembla, Port Jackson and Botany Bay are highly urbanised estuaries with 80–100 years of industrialisation and urbanisation [34], [35], [36]. Port Hacking, The Clyde, Wagonga Inlet and Jervis Bay are estuaries that are relatively less modified by urbanisation and have no history of major industry. In addition, three of our relatively unmodified estuaries (The Clyde, Wagonga Inlet and Jervis Bay) are within Marine Protected Areas where trawling and commercial fishing are restricted [37]. Seven sites (between 1 and 2 km apart) were sampled in each estuary (Figure 1).

Benthic sediments were collected between February and March 2010. Two replicate sediment grabs and water quality measures were collected at each site from 5 m depth using a Van Veen grab to target 5 kg surficial sediments. Each sediment grab was homogenised in a clean tray and sub-sampled for infauna (250 mL) and measures of toxic contaminants (metal and PAH concentrations) (150 g), organic enrichment indicators (sediment Chl-a levels and organic carbon) (150 g) and sediment grain size (50 g). An additional two sediment grabs were taken from sites in 6 of the surveyed estuaries (Port Kembla, Port Jackson, Botany Bay, Port Hacking, Wagonga Inlet and The Clyde) for the experimental deployment (details below). Plasticware used in sediment collection was previously soaked in 5% HNO_3_ for a minimum of 24 h and then rinsed in deionised water (Milli-Q™). All sediments for chemical analyses were kept in the dark on ice for transport to the laboratory and then frozen at −20°C until analysis.

Temperature, salinity, turbidity, pH, oxygen and Chl-a levels in the water column directly above sediment collection sites were measured using a multiprobe (YSI-Sonde 6600-v2, Yellow Springs, USA) calibrated according to the manufacturer's instructions.

### Field experiment sediments deployed in benthic recruitment containers

We used benthic recruitment containers (10 cm diameter ×20 cm height Perspex cylinders) to experimentally investigate the contribution of sediment characteristics to observed patterns by deploying sediments collected during our field survey. Bulk surficial sediment was collected from 5 m depth at 7 sites with unvegetated sediments within 6 estuaries (Port Kembla, Port Jackson, Botany Bay, Port Hacking, Wagonga Inlet and The Clyde). The sediments collected from multiple sites within each estuary were combined and homogenised then sieved (2 mm) to remove shells and larger fauna and flora. This created one sediment homogenate to represent each estuary. The six homogenates represented different sediment characteristics. Homogenate sediments were then divided into 7 replicate sub- samples (0.5 kg), which were applied as a top sediment layer (∼2 cm) to replicate recruitment containers (N = 42). Approximately 1 kg of sand was used to line the bottom of each of the recruitment containers with the homogenate sediments layered on top. Sand was collected from a site in Port Jackson where the chemical composition had been previously characterised as uncontaminated [20]. Recruitment containers were frozen at −20°C for at least 3 mo to defaunate the sediments and remained frozen until deployment where they thawed in situ. For deployment, the recruitment containers were attached to aluminium frames 2.1 m long and deployed at ∼3 m depth (below mean low water spring) in a site previously surveyed within the upper catchment of the Clyde estuary (Figure 1) for 12 weeks (Nov 2010–Feb 2011). Recruitment containers were capped before collection. Surface sediments (∼5 cm depth) from each replicate recruitment container were homogenised in a clean tray and sub-sampled for analyses of infauna, metals and grain size. All sediments for chemical analyses were kept in the dark on ice for transport to the laboratory and then frozen until analysis.

### Sample processing and analysis

Infaunal sub-samples (125-ml) were stained with Rose Bengal and preserved in a 7% formalin solution then passed through a 2 mm mesh (to remove large debris) and onto a 500-µm sieve. The remaining organisms were sorted with a dissecting microscope and identified to the lowest feasible taxonomic level (mostly order). Polychaetes comprised >70% of all assemblages and were therefore identified to family and we used this higher taxonomic resolution to focus on patterns in polychaete family diversity and polychaete abundance in response to anthropogenic modification. A reference collection was deposited at the Australian Museum.

Sediment characteristics were investigated from benthic sediments collected at each site in the survey and analyses included concentrations of major metal and PAH contaminants, total organic carbon and Chl-a and sediment grain size according to the techniques described in [20]. Sediments deployed in recruitment containers were also analysed for metal contaminants and grain sizes at the start (homogenised mixture) and end (each replicate recruitment container) of the experiment.

Sediment metal analyses were undertaken using a microwave-assisted acid digestion according to Method 3051A [38]. The metal concentrations in acid-digests were analysed using inductively coupled plasma-mass spectrometry (ICP-MS; Perkin Elmer, Optima7300DV, USA). As part of the quality assurance, filter and acid-digest blanks, replicates for 20% of samples, analyte sample-spikes and the certified reference materials (CRMs) were measured. Replicates were within 20% and recoveries for spikes and CRMs, (LGC6137, Graham B. Jackson Pty Ltd, Australia National Research Council Canada, NRCC), were within 85–115% of expected values. Full details of recoveries are included in supplementary materials (Table S1). Metal contaminants were included in further statistical analysis if they exceeded the low trigger values from the interim sediment quality guidelines (SQGs) [39]. Cr, Cu, Ni, Pb, Zn exceeded SQGs in the field surveys and Cu, Pb, Zn in the field experiment.

Sixteen PAHs were analysed including naphthalene, acenaphthylene, acenaphthene, fluorene, phenanthrene, anthracene, fluofranthene, pyrene, benz(a)anthracene, chrysene, benzo(a)pyrene, benzo(b)fluoranthene, benzo(k)fluoranthene, indeno(1,2,3-cd)pyrene, dibenzo(a,h)anthracene and benzo(g,h,i)perylene. Analyses of PAHs in sediments followed Method 8260 [40]. Surrogate PAHs (deuterated internal standards; acenaphthene-d_10_, phenanthrene-d_10_, chrysene-d_12_ and perylene-d_12_) were spiked into all samples and recoveries were 111±19%. Individual PAHs were summed for each sample to give a value of total PAHs [39] and this was used in further statistical analyses.

Sediment Chl-a analysis were undertaken following extractions of pigments in acetone with a spectrophotometer (Lambda 35 UV/Vis) according to Greenberg et al. [41]. Inorganic carbon in benthic sediments was removed by acidification with 2 mL of 1 M HCl overnight [42], and total organic carbon (TOC) was analysed using a Leco CN2000 analyser (Leco Corporation, USA) at a combustion temperature of 1050°C. Sediment grain size analyses were made by wet sieving through graded sieves; gravel (2 mm), sand (2 mm–63 µm), and fines (<63 µm). Samples were then oven dried (24 h at 60°C) and weighed to determine the percentage contribution of each fraction.

Porewaters were extracted from the sediments by centrifugation at 800 g for 5 min and then filtered (0.45 µm, Sartorius Minisart) immediately to minimise exposure to air. Samples were taken for ammonia (stored frozen until analyses). Dissolved ammonia was analysed colorimetrically using a Merck Spectroquant Kit (14752).

### Data analysis

In all analyses ‘Modification Category’ (heavily modified or relatively unmodified) was treated as a fixed orthogonal factor and ‘Estuary (within Modification Category)’ was a random nested factor. Replicates in each analysis were either sites (survey) or recruitment containers (experiment). Infaunal community composition was compared between modification categories with permutational multivariate analysis of variance (MANOVA) and Principal Components Analysis (PCO). We also investigated changes in taxa richness and abundance between modification categories because these measures are closely linked to contaminants (both inorganic and organic) [31], [43], and are robust to taxonomic changes when sampling across large spatial scales e.g. estuaries [44]. Also, many other benthic indices have been developed specifically for European monitoring [45] and require a higher level of taxonomic resolution than is often possible with Australian infauna. Polychaete worms comprised more than 50% of the assemblage on average and were the dominant taxon in all samples (Figure S1). Therefore we limited our univariate analysis of ecological measures (taxa richness and abundance) to polychaete families and polychaete individuals. Sediment characteristics (survey and experiment) and water quality variables (survey only) were also compared between modification categories using these models. Infaunal assemblage data included all taxa identified during the survey or experiment and fourth root transformed resemblance matrices were generated with the Bray Curtis dissimilarity index to reflect changes in relative abundances and composition of assemblages. Univariate measures of polychaete abundance and polychaete family richness were also fourth root transformed and environmental variables were normalised. Resemblance matrices for each dataset were then constructed from Euclidean distances. Permutational analysis of variance (ANOVA) of univariate data and permutational multivariate analysis of variance (MANOVA) were performed using type III sums of squares under the reduced model. Homogeneity of dispersion between groups was tested using PERMDISP and no significant dispersion was detected for ‘Modification Category’. Full details of the infauna dataset and analyses are included in supplementary materials (Tables S2, S3, S4, S5, S6).

Principal components analysis (PCA) was used to visualize the entire surveyed infaunal assemblages at each site or in each recruitment container. All analyses were done in PRIMER v6 with PERMANOVA+ [46]. Univariate plots of metals and environmental variables are included in supplementary materials (Figures S2, S3, S4).

## Results

### Field surveys of sediments in multiple estuaries

#### Sediments more contaminated in heavily modified estuaries

Benthic sediments had higher metal (Cr, Cu, Ni, Pb and Zn) and PAH concentrations in heavily modified estuaries than relatively unmodified estuaries (Table 1, Figure S2). Many exceeded SQG trigger values (Cr: 80 µg/g; Cu: 65 µg/g; Ni: 21 µg/g; Pb: 50 µg/g; Zn: 200 µg/g) in the heavily modified estuaries and were often above upper limits (Pb: 220 µg/g; Zn: 410 µg/g), but were never above SQG trigger values in the relatively unmodified estuaries. Metal and PAH concentrations also varied among estuaries, with the exception of chromium (Table 1, Figure S2). Sediment chl-a, porewater ammonia and TOC were elevated in heavily modified estuaries suggesting significant inorganic and organic enrichment of these sediments (Table 1, Figure S2). Sediment grain size was finer for benthic sediment from heavily modified estuaries, indicating a greater capacity for contaminant binding and retention and turbidity levels were also highest in heavily modified estuaries (Table 1, Figure S2). Other water quality variables did not differ between modification categories, but varied significantly among estuaries, with the exception of temperature (Table 1, Figure S3).

**Table 1:** Summary results from permutational ANOVA of sediment characteristics and water quality collected in field surveys of benthic sediments.

Variable	Modification category	Estuary(Mo)
**Sediment characteristics**		
Cr	*	N.S.
Cu	*	**
Ni	*	*
Pb	*	**
Zn	*	**
Total PAHs	**	**
Sediment Chl-a	**	N.S.
Porewater ammonia	**	N.S.
Total organic carbon	N.S.	**
Percent fines	*	N.S.
**Water quality**		
Turbidity	*	**
Salinity	N.S.	**
pH	N.S.	*
Dissolved oxygen	N.S.	*
Water Chl-a	N.S.	*
Temperature	N.S.	N.S.

Factors include Modification category (Mo; heavily modified or relatively unmodified) and Estuary (Es; nested in Modification category). Sites were the replicates (n = 7).

* = p<0.05,

** = p<0.01 and N.S. =  not significant.

#### Infaunal community composition differed in response to modification

**Figure 2:**
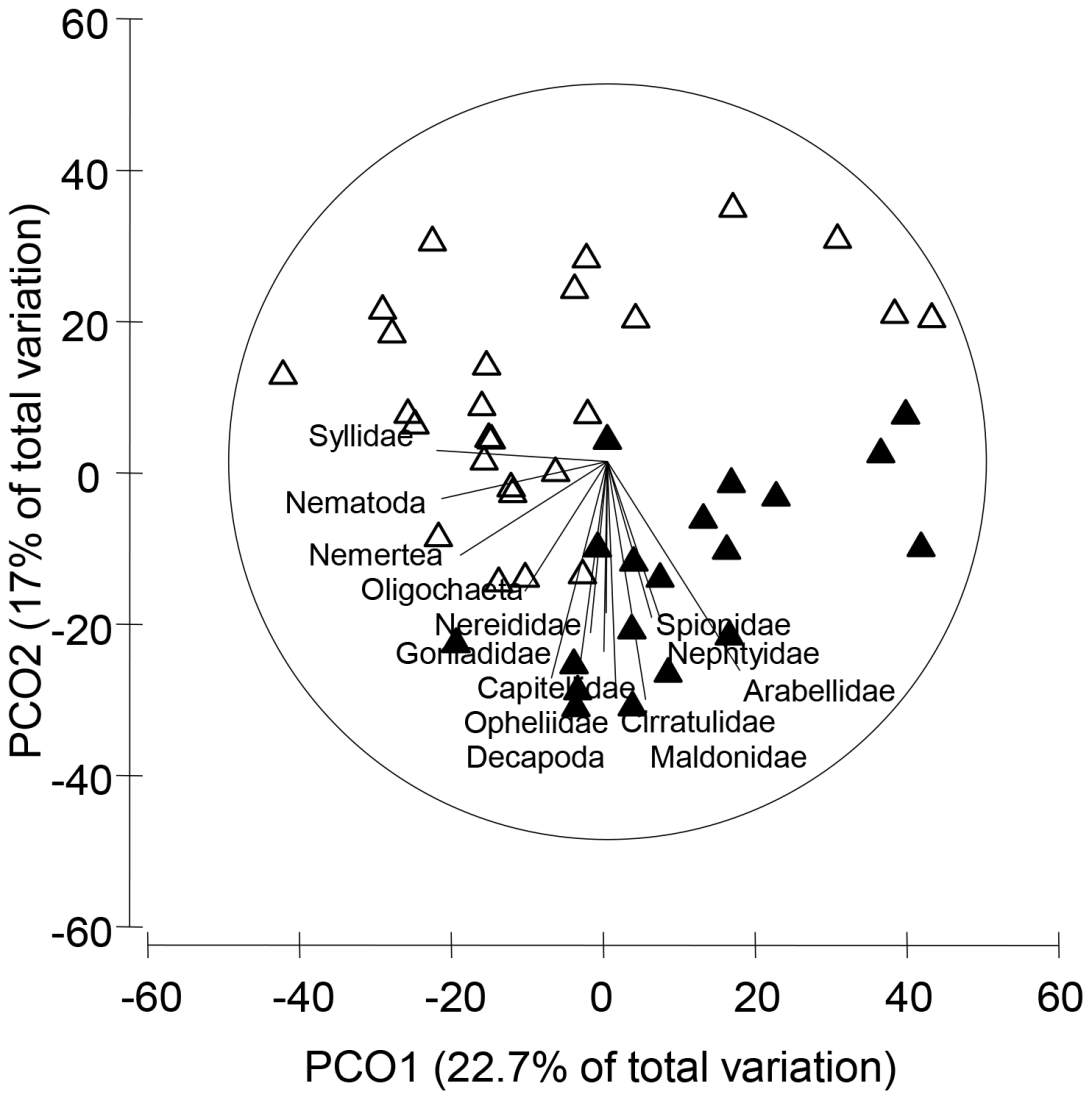
Unconstrained PCA plot of infaunal communities in large-scale field survey. Grabs were sampled from seven heavily modified (filled triangles) or relatively unmodified estuaries (empty triangles). A vector plot of the infaunal community (R>0.4) is also presented to highlight compositional differences among modification categories.

The infaunal community composition differed significantly between heavily modified and relatively unmodified estuaries and also among individual estuaries (Table 2; Figure 2). A combination of the two axes of the unconstrained ordination (PCO) emphasised differences among disturbance categories, although a relatively small proportion (39.7%) of the total variation in the community composition was explained. Sites from heavily modified estuaries (positive scores on PCO1 and negative scores on PCO2) were characterised by greater abundances of individuals from the polychaete families Arabellidae, Spionidae, Nephytidae, Cirratulidae, Maldanidae and Capitellidae (Figure 2). Representatives of the family Syllidae were more abundant in relatively unmodified estuaries (negative scores on PCO1 and positive scores on PCO2).

**Table 2:** Results of permutational MANOVA of (a) the entire infaunal community, and univariate ANOVA of (b) polychaete family richness and (c) polychaete abundance from field surveys of benthic sediments.

Source	df	SS	MS	Pseudo-F	P(perm)
		(a) Infaunal community composition			
Modification category	1	10341	10341	2.04	**0.018**
Estuary (Mo)	5	25372	5074	4.04	**0.000**
Res	39	48951	1255		
		(b) Polychaete family richness			
Modification category	1	391.69	391.69	10.67	**0.044**
Estuary (Mo)	5	183.40	36.68	7.74	**0.001**
Res	39	184.81	4.74		
		(c) Polychaete abundance			
Modification category	1	1610	1610	8.28	**0.040**
Estuary (Mo)	5	971	194	1.47	0.218
Res	39	5144	132		
					

Factors include Modification category (Mo; heavily modified or relatively unmodified) and Estuary (Es; nested in Modification category). Sites were the replicates (n = 7).

#### Polychaetes more diverse and abundant in heavily modified estuaries

**Figure 3:**
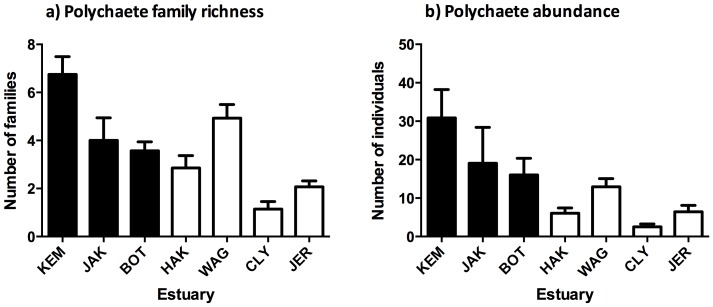
Polychaete family richness and abundance in large-scale field survey. Mean (+S.E.) polychaete (a) family richness and (b) abundance (number of individuals) in benthic sediments collected from seven sites in large-scale field surveys of seven heavily modified (filled bars) or relatively unmodified (open bars) estuaries.

Polychaete richness (number of families) and abundance (number of individuals) differed significantly between modification categories (Figure 3; Table 2). Heavily modified estuaries supported a more diverse and abundant polychaete community than relatively unmodified estuaries.

In summary, sediments from heavily modified estuaries were more contaminated with metals and PAHs and organically enriched than the relatively unmodified estuaries. These measures of toxic and enriching contamination also varied between estuaries Grain sizes were finer in heavily modified estuaries; conversely most variation in water quality was between estuaries rather than between heavily modified and relatively unmodified estuaries. Therefore differences in the infaunal community and measures of polychaete richness and abundance with modification category are likely to be related to contamination or sediment grain size. Infaunal differences between estuaries could be related to metal concentrations, sediment grain size or environmental variables.

### Field experiment sediments deployed in benthic recruitment containers

**Figure 4:**
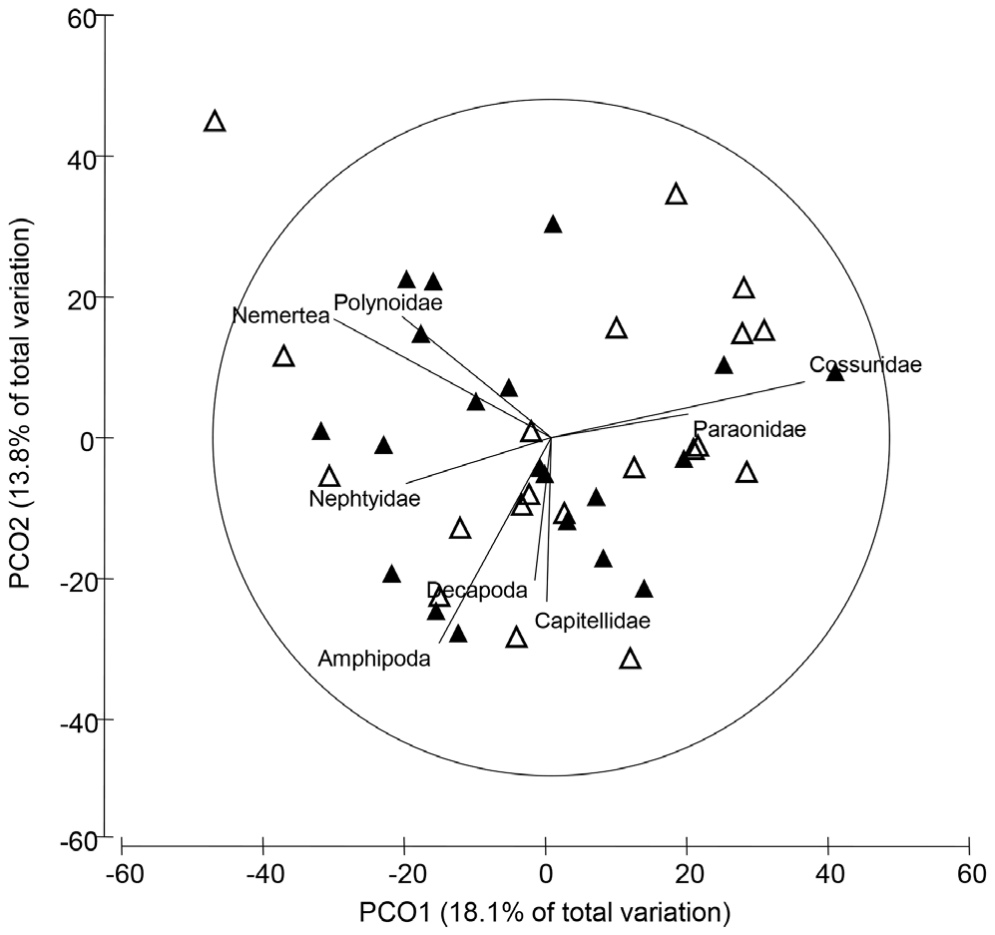
Unconstrained PCA plot of infaunal communities in field manipulated sediment. Sediment was sourced from six estuaries that were either heavily modified (filled triangles) or relatively unmodified estuaries (empty triangles), and deployed in benthic recruitment containers in the Clyde. A vector plot of the infaunal community (R>0.4) is also presented to highlight compositional differences among modification categories.

**Figure 5:**
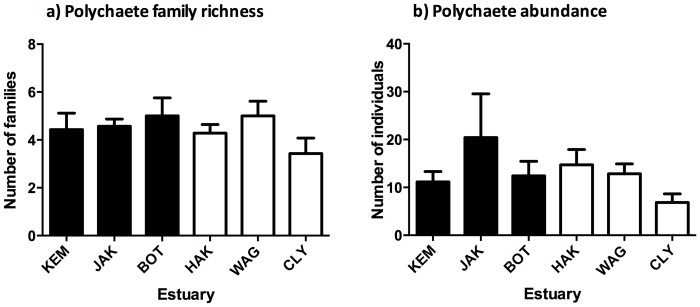
Polychaete family richness and abundance in field manipulated sediments. Mean (+S.E.) polychaete (a) family richness and (b) abundance (number of individuals) in field manipulated sediment sourced from six heavily modified (filled bars) or relatively unmodified (open bars) estuaries and deployed in the Clyde.

Characteristics (including metals and grain sizes) of the field-collected sediments differed consistently between modification categories at the start and the end of the field experiment (Figure S4, Table S6). Additionally since the source sediments had increased infaunal abundances (Figure 3; Table 2) they likely experienced more organic loading from the breakdown of these infauna upon deployment. Despite these differences in the sediment characteristics, infaunal community composition did not differ between modification category or source estuary sediment (Figure 4; Table 3). Furthermore, when polychaete ecological measures were analysed with a similar statistical design to the field survey, richness and abundance did not differ between source sediments (Figure 5; Table 3). Patterns of increased polychaete richness and abundance that were observed in highly contaminated sediments in the field survey were not present in the deployed sediments.

**Table 3:** Results of permutational MANOVA of (a) the entire infaunal community, and permutational ANOVA of (b) polychaete family richness and (c) polychaete abundance from field transplant experiments of benthic sediments.

Source	df	SS	MS	F	P(perm)
		(a) Infaunal community composition			
Modification category	1	2417.7	2417.7	0.77	0.652
Estuary (Mo)	4	12623	3155.9	1.42	0.061
Res	36	75425	2218.4		
		(b) Polychaete family richness			
Modification category	1	0.02	0.02	0.81	0.423
Estuary (Mo)	4	0.10	0.02	1.24	0.310
Res	36	0.69	0.02		
		(c) Polychaete abundance			
Modification category	1	106.88	106.88	0.72	0.436
Estuary (Mo)	4	590.48	147.62	1.10	0.373
Res	36	4837.40	134.37		
					

Factors include Modification category (Mo; heavily modified or relatively unmodified), and Estuary (Es; nested in Modification category). BRCs were the replicates (n = 7).

## Discussion

Most national biomonitoring guidelines recommend the use of multiple lines of evidence when assessing environmental impacts including the use of experiments to establish causation [39], [47], [48], [49], [50]. Environmental monitoring studies, however, rarely use manipulative field experiments to interrogate the patterns found in observational studies. This may result in the misinterpretation of monitoring data and an inability to differentiate the effects of multiple co-varying stressors [26]. We investigated infaunal community composition and contaminant measures in a large-scale survey of benthic sediments. Based on previous research (meta-analysis by [30]) we expected to see clear ‘negative’ effects of toxic contaminants in heavily modified estuaries (e.g. decreases in abundance and diversity). However, we found that polychaetes in particular were more diverse and abundant in heavily modified estuaries. Sediment Chl-a, porewater ammonia and total organic carbon levels co-varied with toxic contaminants and were also elevated in heavily modified estuaries; we therefore hypothesised that organic enrichment of the sediments may have been driving the observed patterns [31]. Observations from the survey were further investigated with a field experiment that determined the extent to which the patterns were caused by small-scale sediment characteristics or other large-scale environmental conditions that co-vary with anthropogenic modification and disturbance (e.g. organic enrichment and the local species pool). Our field experiment deployed sediments from the surveyed estuaries at one relatively uncontaminated location. Recruitment during the 12 week experiment was subject to local conditions and in this situation we no longer observed elevated polychaete diversity in sediments from heavily modified estuaries. This suggests that estuarine conditions will play a major role in determining benthic community diversity, irrespective of small-scale sediment characteristics.

Habitats that are severely stressed by anthropogenic activities are reported to have substantially reduced species richness (reviewed in [30]) and often support only a few opportunistic or tolerant species [31]. Laboratory and field studies have found infaunal abundances to decrease with increasing metal (Cu) concentrations [51], [52] and sediment Cu concentrations measured from estuaries in the current study were among the highest recorded globally in impacted estuaries [53], [54], [55]. Despite severe metal concentrations far above guideline values up to 1250 ug/g Cu, 550 ug/g Pb and 900 ug/g Zn (Figure S1), we found the opposite for polychaete assemblages, with abundance and family richness higher in heavily modified estuaries. This suggests that toxic effects of contaminants were not significant and may reflect lower bioavailability of contaminants due to increased binding with silt and organic material in heavily modified estuaries [56]. Furthermore, past studies have found some taxa (e.g. polychaetes) to contain metal tolerant species in both hard substrate and soft sediment communities [51], [57], [58]. However, more commonly, elevated species richness and abundance in heavily contaminated areas have been associated with organic enrichment [59], [60], [61].

Most modified estuaries are exposed to a range of inputs (e.g. urban or agricultural runoff or sewage) that would enrich the nutrients within the estuary and may increase the diversity of the local species pool [62]. We found evidence of enhanced sediment Chl-a and high levels of TOC in sediments from heavily modified estuaries suggesting high levels of organic enrichment. Data collected in a separate study also indicated our heavily modified estuaries have higher organic loading than the relatively unmodified estuaries [63]. Organic enrichment from sources such as sewage has been associated with increases in infaunal abundances and diversity [31], which may go some way in explaining the responses we observed in the diversity and abundance of polychaete assemblages from heavily modified estuaries (in particular the surface deposit feeding families; Capitellidae and Cirratulidae). It is important to note that estuaries in NSW are relatively oligotrophic, with nutrient loads an order of magnitude less than estuaries in other parts of the world [64]. Therefore our “heavily modified” estuaries may reflect a state of more moderate pollution where we might expect this increased diversity to be temporary and due to tolerant species [23], [31] rather than a “positive” effect of nutrient addition. Estuaries with higher nutrient loads can be subject to severe eutrophication that negatively impacts polychaete diversity and eventually abundance [65], [66].

However, large-scale anthropogenic impacts may not be reflected in smaller-scale manipulations [67], [68]. In our survey across multiple estuaries we found that polychaete richness and abundance differed among relatively pristine and modified estuaries, however, we found that in experimental deployments of sediment, these ecological measures did not differ between modification category or source estuary. This suggests that there is no small-scale effect of sediment characteristics (even severe metal or organic contamination) because organic enrichment at the scale of an entire estuary is having a greater effect on infaunal diversity and abundance. Our findings agree with recent work [69], [70] where continual organic enrichment from farming processes resulted in increased macrofaunal abundances despite expectations of negative impacts from this contamination. However, the apparent lack of sediment metal effects on the experimental community contrasts with recent field mesocosm work [71] in which responses to metal concentrations were evident. Differences in effects may also be related to the scale of the study as their deployments were in multiple estuaries, and reciprocal transplants may be the way forward to optimise the usefulness of manipulative experiments. Temporal scale may have also influenced differences between the results of our experiment and the survey since our surveyed assemblages were well established and our recruitment containers were deployed for only 12 weeks. This period may not have allowed enough time for the initial acclimation of the sediments with respect to their chemistry [72], and subsequent infaunal recruitment before they were collected again and processed. Changes in contaminant concentrations and sediment loss through resuspension are important considerations for experimental field manipulations of sediments [72]. Metal concentrations decreased during the experiment, but were still highest in the heavily modified sediments at the end of the study. However, studies have demonstrated rapid recolonisation of defaunated sediments [73] and other experimental deployments of sediments have found this period of time to be sufficient to detect effects of similar sediment treatments [74]. Furthermore the number of families and abundances were similar to the observational study results at that site or higher in the experiment indicating that the assemblage had possibly reached equilibrium.

## Conclusion

The outcomes of this study have relevance for environmental managers as the patterns we observed across regional scales provided important information about the ecological condition of estuaries and useful directions for future research. The scale of field manipulations may also be an important consideration, as the patterns of increased polychaete richness we observed in heavily modified estuaries across regional scales were not found in our small-scale manipulations within one estuary. This suggests that increases in diversity are related to parameters that co-vary with estuary modification over larger scales such as the productivity of the entire system and the diversity of the regional species pool, and patches of contaminated sediments will not result in similar effects. If environmental managers rely solely on small-scale surveys or manipulations together with meta-analyses from the literature this may lead to a poor understanding of actual impacts over regional scales. Ecological monitoring of human impacts should, as a matter of course, use multiple lines of evidence including field surveys and field experiments in order to reliably identify the drivers of biological patterns and initial observational studies can inform the design of manipulative experiments to further investigate causation.

## Supporting Information

Figure S1 Mean (+S.E.) proportional abundances of different taxa analysed from benthic sediment grabs collected in seven estuaries.
(DOCX)

Figure S2 Mean (+S.E.) metal and total PAH concentrations (dw), sediment Chl-a, TOC, porewater ammonia and percent fines (<63 µm) analysed from benthic sediment grabs collected in heavily modified (filled bars) and relatively unmodified (open bars) estuaries.
(DOCX)

Figure S3 Mean (+S.E) values for water quality variables (salinity, temperature, pH, turbidity, dissolved oxygen and chlorophyll-a) collected in heavily modified (filled bars) and relatively unmodified (open bars) estuaries.
(DOCX)

Figure S4 Mean (+S.E.) end metal concentrations (dw) and percent fines (<63 µm) analysed from deployed sediments sourced from heavily modified (filled bars) and relatively unmodified (open bars) estuaries. Starting concentrations are indicated as a single figure above the bars.
(DOCX)

Table S1 Details of limits of detection for each of the analytes sampled from sediments.
(DOCX)

Table S2 Soft sediment infauna sampled in benthic sediment surveyed from seven sites in seven NSW estuaries that were either heavily modified or relatively unmodified.
(DOCX)

Table S3 Soft sediment infauna sampled in field manipulated sediment.
(DOCX)

Table S4 Permutational ANOVA results for sediment quality measures in benthic sediment surveyed from seven NSW estuaries.
(DOCX)

Table S5 Permutational ANOVA results for water quality variables surveyed from seven NSW estuaries.
(DOCX)

Table S6 Permutational ANOVA results for metal contaminants and sediment quality variables measured at the start and end in sediment field experiments deployed in the Clyde estuary.
(DOCX)
