# Supplementary material for: Polychaete Richness and Abundance Enhanced in Anthropogenically Modified Estuaries Despite High Concentrations of Toxic Contaminants
Source: PLoS One. 2013 Sep 30;8(9):e77018. doi: 10.1371/journal.pone.0077018 (PMC3786951; doi:10.1371/journal.pone.0077018)
Supplement: Figure S1 — Mean (+S.E.) proportional abundances of different taxa analysed from benthic sediment grabs collected in seven estuaries. (DOCX) [file pone.0077018.s001.docx]

**Figure S1.** Mean (+S.E.) proportional abundances of different taxa analysed from benthic sediment grabs collected in seven estuaries.
