# Supplementary material for: Polychaete Richness and Abundance Enhanced in Anthropogenically Modified Estuaries Despite High Concentrations of Toxic Contaminants
Source: PLoS One. 2013 Sep 30;8(9):e77018. doi: 10.1371/journal.pone.0077018 (PMC3786951; doi:10.1371/journal.pone.0077018)
Supplement: Figure S2 — Mean (+S.E.) metal and total PAH concentrations (dw), sediment Chl-a, TOC, porewater ammonia and percent fines (<63 µm) analysed from benthic sediment grabs collected in heavily modified (filled bars) and relatively unmodified (open bars) estuaries. (DOCX) [file pone.0077018.s002.docx]

**Figure S2.** Mean (+S.E.) metal and total PAH concentrations (dw), sediment Chl-a, TOC, porewater ammonia and percent fines (<63 µm) analysed from benthic sediment grabs collected in heavily modified (filled bars) and relatively unmodified (open bars) estuaries.
