# Supplementary material for: Polychaete Richness and Abundance Enhanced in Anthropogenically Modified Estuaries Despite High Concentrations of Toxic Contaminants
Source: PLoS One. 2013 Sep 30;8(9):e77018. doi: 10.1371/journal.pone.0077018 (PMC3786951; doi:10.1371/journal.pone.0077018)
Supplement: Figure S3 — Mean (+S.E) values for water quality variables (salinity, temperature, pH, turbidity, dissolved oxygen and chlorophyll-a) collected in heavily modified (filled bars) and relatively unmodified (open bars) estuaries. (DOCX) [file pone.0077018.s003.docx]

**Figure S3.** Mean (+S.E) values for water quality variables (salinity, temperature, pH, turbidity, dissolved oxygen and chlorophyll-a) collected in heavily modified (filled bars) and relatively unmodified (open bars) estuaries.
