# Supplementary material for: Polychaete Richness and Abundance Enhanced in Anthropogenically Modified Estuaries Despite High Concentrations of Toxic Contaminants
Source: PLoS One. 2013 Sep 30;8(9):e77018. doi: 10.1371/journal.pone.0077018 (PMC3786951; doi:10.1371/journal.pone.0077018)
Supplement: Figure S4 — Mean (+S.E.) end metal concentrations (dw) and percent fines (<63 µm) analysed from deployed sediments sourced from heavily modified (filled bars) and relatively unmodified (open bars) estuaries. Starting concentrations are indicated as a single figure above the bars. (DOCX) [file pone.0077018.s004.docx]

**Figure S4.** Mean (+S.E.) end metal concentrations (dw) and percent fines (<63 µm) analysed from deployed sediments sourced from heavily modified (filled bars) and relatively unmodified (open bars) estuaries. Starting concentrations are indicated as a single figure above the bars.
