# Supplementary material for: Polychaete Richness and Abundance Enhanced in Anthropogenically Modified Estuaries Despite High Concentrations of Toxic Contaminants
Source: PLoS One. 2013 Sep 30;8(9):e77018. doi: 10.1371/journal.pone.0077018 (PMC3786951; doi:10.1371/journal.pone.0077018)
Supplement: Table S1 — Details of limits of detection for each of the analytes sampled from sediments. (DOCX) [file pone.0077018.s005.docx]

**Table S1.** Details of limits of detection for each of the analytes sampled from sediments.

|  | **LODs** |
| --- | --- |
|  |  |
| **Cr** | 2 mg/kg |
| **Cu** | 7 mg/kg |
| **Ni** | 3 mg/kg |
| **Pb** | 6 mg/kg |
| **Zn** | 2 mg/kg |
| **PAHs** | 0.01-0.02 mg/kg |
| **Chlorophyll-a** | 0.5-4 nm |
| **TOC** | 1 mg/kg |
| **NH_3_** | 0.1 mgNH_3_-N/L |
|  |  |
