# Supplementary material for: Polychaete Richness and Abundance Enhanced in Anthropogenically Modified Estuaries Despite High Concentrations of Toxic Contaminants
Source: PLoS One. 2013 Sep 30;8(9):e77018. doi: 10.1371/journal.pone.0077018 (PMC3786951; doi:10.1371/journal.pone.0077018)
Supplement: Table S2 — Soft sediment infauna sampled in benthic sediment surveyed from seven sites in seven NSW estuaries that were either heavily modified or relatively unmodified. (DOCX) [file pone.0077018.s006.docx]

**Table S2.** Soft sediment infauna sampled in benthic sediment surveyed from seven sites in seven NSW estuaries that were either heavily modified or relatively unmodified. Modification category (Mo; heavily modified (HM) or relatively unmodified (RUM)) and Estuary (Es; nested in Modification category). Sites were the replicates.

| **Mo** | **Es** | **Si** | **Ampharetidae** | **Arabellidae** | **Capitellidae** | **Chaetopteridae** | **Cirratulidae** | **Cossuridae** | **Dorvilleidae** | **Eunicidae** | **Glyceridae** |
| --- | --- | --- | --- | --- | --- | --- | --- | --- | --- | --- | --- |
| HM | KEM | 8 | 0 | 2 | 2 | 0 | 4 | 3 | 0 | 0 | 0 |
| HM | KEM | 9 | 0 | 1 | 11 | 0 | 6 | 0 | 0 | 0 | 0 |
| HM | KEM | 11 | 0 | 0 | 1 | 0 | 0 | 0 | 0 | 0 | 0 |
| HM | KEM | 12 | 0 | 0 | 2 | 0 | 4 | 0 | 0 | 1 | 0 |
| HM | KEM | 13 | 0 | 1 | 2 | 0 | 2 | 0 | 0 | 0 | 0 |
| HM | KEM | 14 | 0 | 1 | 10 | 1 | 6 | 0 | 1 | 0 | 0 |
| HM | JAK | 8 | 0 | 1 | 57 | 0 | 9 | 1 | 0 | 0 | 1 |
| HM | JAK | 9 | 0 | 0 | 7 | 0 | 0 | 0 | 0 | 0 | 0 |
| HM | JAK | 10 | 0 | 1 | 1 | 0 | 0 | 0 | 0 | 0 | 0 |
| HM | JAK | 11 | 0 | 1 | 1 | 0 | 0 | 0 | 0 | 0 | 0 |
| HM | JAK | 12 | 0 | 0 | 0 | 1 | 1 | 0 | 0 | 0 | 0 |
| HM | JAK | 13 | 0 | 3 | 8 | 0 | 2 | 1 | 0 | 0 | 1 |
| HM | JAK | 14 | 0 | 0 | 2 | 0 | 0 | 0 | 0 | 0 | 0 |
| HM | BOT | 8 | 0 | 1 | 1 | 0 | 0 | 0 | 0 | 0 | 0 |
| HM | BOT | 9 | 0 | 1 | 0 | 0 | 0 | 1 | 0 | 0 | 0 |
| HM | BOT | 10 | 0 | 1 | 1 | 0 | 1 | 0 | 0 | 0 | 0 |
| HM | BOT | 11 | 0 | 0 | 1 | 0 | 0 | 1 | 0 | 0 | 0 |
| HM | BOT | 12 | 0 | 0 | 2 | 0 | 0 | 0 | 0 | 0 | 0 |
| HM | BOT | 13 | 1 | 0 | 7 | 0 | 0 | 0 | 0 | 0 | 0 |
| HM | BOT | 14 | 1 | 2 | 4 | 0 | 0 | 0 | 0 | 0 | 0 |

| **Mo** | **Es** | **Si** | **Goniadidae** | **Hesionidae** | **Lumbrineridae** | **Magelonidae** | **Maldonidae** | **Nephtyidae** | **Nereididae** | **Opheliidae** | **Orbiniidae** |
| --- | --- | --- | --- | --- | --- | --- | --- | --- | --- | --- | --- |
| HM | KEM | 8 | 0 | 0 | 0 | 0 | 1 | 0 | 0 | 2 | 1 |
| HM | KEM | 9 | 0 | 0 | 0 | 0 | 1 | 1 | 4 | 2 | 3 |
| HM | KEM | 11 | 0 | 1 | 0 | 0 | 1 | 0 | 0 | 0 | 0 |
| HM | KEM | 12 | 1 | 1 | 1 | 0 | 2 | 1 | 1 | 2 | 0 |
| HM | KEM | 13 | 0 | 2 | 0 | 0 | 0 | 0 | 1 | 2 | 1 |
| HM | KEM | 14 | 0 | 1 | 0 | 0 | 1 | 0 | 0 | 2 | 4 |
| HM | JAK | 8 | 1 | 1 | 0 | 0 | 0 | 0 | 1 | 6 | 0 |
| HM | JAK | 9 | 1 | 0 | 0 | 0 | 0 | 0 | 0 | 0 | 0 |
| HM | JAK | 10 | 0 | 0 | 0 | 0 | 0 | 0 | 1 | 0 | 0 |
| HM | JAK | 11 | 0 | 0 | 0 | 0 | 1 | 1 | 0 | 0 | 0 |
| HM | JAK | 12 | 0 | 0 | 0 | 0 | 0 | 0 | 1 | 0 | 0 |
| HM | JAK | 13 | 0 | 2 | 0 | 0 | 1 | 2 | 0 | 2 | 0 |
| HM | JAK | 14 | 0 | 0 | 0 | 0 | 0 | 1 | 1 | 0 | 0 |
| HM | BOT | 8 | 0 | 0 | 0 | 11 | 0 | 0 | 0 | 0 | 0 |
| HM | BOT | 9 | 0 | 0 | 0 | 2 | 0 | 0 | 0 | 0 | 0 |
| HM | BOT | 10 | 0 | 0 | 0 | 0 | 1 | 0 | 1 | 0 | 0 |
| HM | BOT | 11 | 0 | 0 | 0 | 10 | 0 | 0 | 0 | 0 | 1 |
| HM | BOT | 12 | 0 | 0 | 0 | 0 | 0 | 0 | 0 | 0 | 1 |
| HM | BOT | 13 | 0 | 0 | 3 | 1 | 0 | 0 | 0 | 0 | 0 |
| HM | BOT | 14 | 0 | 0 | 0 | 7 | 0 | 0 | 0 | 0 | 0 |

| **Mo** | **Es** | **Si** | **Paraonidae** | **Phyllodocidae** | **Polynoidae** | **Sabellidae** | **Scalibregmidae** | **Sigalionidae** | **Spionidae** | **Syllidae** | **Terebellidae** |
| --- | --- | --- | --- | --- | --- | --- | --- | --- | --- | --- | --- |
| HM | KEM | 8 | 0 | 0 | 0 | 3 | 0 | 0 | 9 | 0 | 0 |
| HM | KEM | 9 | 3 | 1 | 0 | 1 | 0 | 0 | 32 | 0 | 0 |
| HM | KEM | 11 | 0 | 0 | 0 | 0 | 0 | 0 | 2 | 0 | 0 |
| HM | KEM | 12 | 0 | 0 | 0 | 0 | 0 | 0 | 33 | 1 | 0 |
| HM | KEM | 13 | 0 | 0 | 0 | 1 | 0 | 0 | 3 | 1 | 0 |
| HM | KEM | 14 | 0 | 0 | 0 | 0 | 2 | 0 | 8 | 0 | 0 |
| HM | JAK | 8 | 0 | 0 | 0 | 0 | 0 | 0 | 8 | 2 | 0 |
| HM | JAK | 9 | 0 | 0 | 0 | 0 | 0 | 0 | 0 | 0 | 0 |
| HM | JAK | 10 | 0 | 0 | 0 | 0 | 0 | 2 | 6 | 0 | 0 |
| HM | JAK | 11 | 0 | 1 | 0 | 0 | 0 | 0 | 0 | 0 | 0 |
| HM | JAK | 12 | 0 | 0 | 0 | 0 | 0 | 0 | 1 | 0 | 0 |
| HM | JAK | 13 | 0 | 0 | 0 | 0 | 0 | 1 | 2 | 1 | 0 |
| HM | JAK | 14 | 0 | 0 | 0 | 0 | 0 | 1 | 0 | 0 | 0 |
| HM | BOT | 8 | 0 | 0 | 0 | 0 | 0 | 0 | 1 | 0 | 0 |
| HM | BOT | 9 | 0 | 0 | 0 | 0 | 0 | 0 | 14 | 0 | 0 |
| HM | BOT | 10 | 0 | 0 | 0 | 0 | 0 | 0 | 0 | 0 | 0 |
| HM | BOT | 11 | 0 | 0 | 0 | 1 | 0 | 0 | 1 | 0 | 0 |
| HM | BOT | 12 | 0 | 0 | 0 | 0 | 0 | 0 | 1 | 1 | 0 |
| HM | BOT | 13 | 0 | 0 | 1 | 1 | 0 | 0 | 6 | 2 | 0 |
| HM | BOT | 14 | 0 | 0 | 0 | 0 | 0 | 0 | 29 | 0 | 1 |

| **Mo** | **Es** | **Si** | **Trichobranchidae** | **Juveniles** | **Oligochaeta** | **Nematoda** | **Nemertea** | **Sipuncula** | **Priapulida** | **Phoronida** | **Amphipoda** |
| --- | --- | --- | --- | --- | --- | --- | --- | --- | --- | --- | --- |
| HM | KEM | 8 | 0 | 0 | 1 | 1 | 3 | 0 | 0 | 0 | 0 |
| HM | KEM | 9 | 0 | 0 | 0 | 3 | 1 | 0 | 0 | 0 | 0 |
| HM | KEM | 11 | 0 | 0 | 0 | 1 | 0 | 0 | 0 | 0 | 1 |
| HM | KEM | 12 | 0 | 0 | 2 | 22 | 0 | 0 | 0 | 0 | 1 |
| HM | KEM | 13 | 0 | 0 | 1 | 2 | 3 | 1 | 0 | 0 | 3 |
| HM | KEM | 14 | 0 | 0 | 2 | 22 | 0 | 0 | 0 | 0 | 5 |
| HM | JAK | 8 | 0 | 0 | 0 | 6 | 1 | 1 | 0 | 0 | 0 |
| HM | JAK | 9 | 0 | 0 | 5 | 6 | 2 | 0 | 0 | 0 | 0 |
| HM | JAK | 10 | 0 | 0 | 0 | 0 | 0 | 0 | 0 | 0 | 0 |
| HM | JAK | 11 | 0 | 0 | 1 | 2 | 1 | 1 | 0 | 0 | 1 |
| HM | JAK | 12 | 0 | 0 | 1 | 2 | 1 | 0 | 0 | 1 | 0 |
| HM | JAK | 13 | 1 | 0 | 2 | 0 | 5 | 3 | 0 | 0 | 1 |
| HM | JAK | 14 | 0 | 0 | 1 | 0 | 0 | 0 | 0 | 0 | 1 |
| HM | BOT | 8 | 0 | 0 | 0 | 1 | 0 | 0 | 0 | 0 | 0 |
| HM | BOT | 9 | 0 | 0 | 0 | 2 | 0 | 0 | 0 | 0 | 0 |
| HM | BOT | 10 | 0 | 0 | 0 | 0 | 1 | 0 | 0 | 0 | 0 |
| HM | BOT | 11 | 0 | 0 | 0 | 0 | 4 | 0 | 0 | 0 | 0 |
| HM | BOT | 12 | 0 | 0 | 0 | 16 | 9 | 0 | 0 | 0 | 15 |
| HM | BOT | 13 | 0 | 0 | 1 | 10 | 0 | 0 | 0 | 0 | 0 |
| HM | BOT | 14 | 0 | 0 | 0 | 0 | 1 | 0 | 0 | 0 | 0 |

| **Mo** | **Es** | **Si** | **Copepoda** | **Isopoda** | **Ostrocoda** | **Tanaidacea** | **Cumacea** | **Decapoda** | **Brachyura** | **Bivalvia** | **Gastropoda** | **Echinoidea** | **Asteroidea** |
| --- | --- | --- | --- | --- | --- | --- | --- | --- | --- | --- | --- | --- | --- |
| HM | KEM | 8 | 0 | 0 | 0 | 0 | 0 | 0 | 0 | 1 | 0 | 0 | 0 |
| HM | KEM | 9 | 0 | 1 | 0 | 0 | 0 | 1 | 0 | 0 | 1 | 0 | 0 |
| HM | KEM | 11 | 0 | 0 | 0 | 0 | 0 | 0 | 0 | 0 | 0 | 0 | 0 |
| HM | KEM | 12 | 0 | 1 | 1 | 1 | 0 | 1 | 0 | 0 | 0 | 0 | 0 |
| HM | KEM | 13 | 0 | 0 | 0 | 1 | 0 | 0 | 0 | 1 | 0 | 0 | 0 |
| HM | KEM | 14 | 1 | 1 | 0 | 2 | 0 | 2 | 0 | 1 | 0 | 0 | 0 |
| HM | JAK | 8 | 1 | 0 | 0 | 0 | 0 | 2 | 0 | 0 | 0 | 0 | 0 |
| HM | JAK | 9 | 0 | 0 | 0 | 0 | 0 | 1 | 0 | 0 | 0 | 0 | 0 |
| HM | JAK | 10 | 0 | 1 | 0 | 0 | 0 | 0 | 0 | 0 | 0 | 0 | 0 |
| HM | JAK | 11 | 0 | 0 | 1 | 0 | 0 | 0 | 0 | 1 | 0 | 0 | 0 |
| HM | JAK | 12 | 0 | 0 | 0 | 0 | 1 | 0 | 0 | 0 | 0 | 0 | 0 |
| HM | JAK | 13 | 0 | 0 | 0 | 0 | 0 | 1 | 0 | 0 | 0 | 0 | 0 |
| HM | JAK | 14 | 0 | 0 | 0 | 0 | 0 | 0 | 0 | 0 | 0 | 0 | 0 |
| HM | BOT | 8 | 0 | 0 | 0 | 0 | 0 | 0 | 0 | 0 | 0 | 0 | 0 |
| HM | BOT | 9 | 1 | 0 | 0 | 0 | 0 | 0 | 0 | 1 | 0 | 0 | 0 |
| HM | BOT | 10 | 1 | 0 | 0 | 0 | 0 | 0 | 0 | 0 | 0 | 0 | 0 |
| HM | BOT | 11 | 0 | 0 | 0 | 0 | 0 | 0 | 0 | 1 | 0 | 0 | 0 |
| HM | BOT | 12 | 2 | 1 | 0 | 0 | 1 | 0 | 0 | 0 | 1 | 0 | 0 |
| HM | BOT | 13 | 2 | 0 | 0 | 0 | 1 | 0 | 0 | 2 | 0 | 0 | 0 |
| HM | BOT | 14 | 0 | 0 | 0 | 0 | 0 | 0 | 0 | 1 | 0 | 0 | 0 |

| **Mo** | | **Es** | | | **Si** | | | | **Ampharetidae** | | | | | **Arabellidae** | | | | | **Capitellidae** | | | | | **Chaetopteridae** | | | | | | | **Cirratulidae** | | | | **Cossuridae** | | | | | **Dorvilleidae** | | | | | **Eunicidae** | | | | | **Glyceridae** | | | |  |  |
| --- | --- | --- | --- | --- | --- | --- | --- | --- | --- | --- | --- | --- | --- | --- | --- | --- | --- | --- | --- | --- | --- | --- | --- | --- | --- | --- | --- | --- | --- | --- | --- | --- | --- | --- | --- | --- | --- | --- | --- | --- | --- | --- | --- | --- | --- | --- | --- | --- | --- | --- | --- | --- | --- | --- | --- |
| RUM | | HAK | | | 15 | | | | 0 | | | | | 0 | | | | | 0 | | | | | 0 | | | | | | | 0 | | | | 0 | | | | | 0 | | | | | 0 | | | | | 0 | | | |  |  |
| RUM | | HAK | | | 17 | | | | 0 | | | | | 0 | | | | | 0 | | | | | 0 | | | | | | | 1 | | | | 0 | | | | | 0 | | | | | 0 | | | | | 0 | | | |  |  |
| RUM | | HAK | | | 19 | | | | 0 | | | | | 0 | | | | | 0 | | | | | 0 | | | | | | | 0 | | | | 0 | | | | | 0 | | | | | 0 | | | | | 0 | | | |  |  |
| RUM | | HAK | | | 20 | | | | 0 | | | | | 0 | | | | | 0 | | | | | 0 | | | | | | | 0 | | | | 0 | | | | | 0 | | | | | 0 | | | | | 0 | | | |  |  |
| RUM | | HAK | | | 25 | | | | 0 | | | | | 0 | | | | | 1 | | | | | 0 | | | | | | | 0 | | | | 1 | | | | | 0 | | | | | 0 | | | | | 0 | | | |  |  |
| RUM | | HAK | | | 26 | | | | 0 | | | | | 0 | | | | | 0 | | | | | 0 | | | | | | | 1 | | | | 0 | | | | | 0 | | | | | 0 | | | | | 0 | | | |  |  |
| RUM | | HAK | | | 27 | | | | 0 | | | | | 0 | | | | | 0 | | | | | 0 | | | | | | | 1 | | | | 0 | | | | | 0 | | | | | 0 | | | | | 0 | | | |  |  |
| RUM | | WAG | | | 8 | | | | 0 | | | | | 0 | | | | | 6 | | | | | 0 | | | | | | | 0 | | | | 0 | | | | | 0 | | | | | 0 | | | | | 0 | | | |  |  |
| RUM | | WAG | | | 9 | | | | 0 | | | | | 0 | | | | | 9 | | | | | 0 | | | | | | | 0 | | | | 0 | | | | | 1 | | | | | 0 | | | | | 0 | | | |  |  |
| RUM | | WAG | | | 10 | | | | 0 | | | | | 0 | | | | | 4 | | | | | 0 | | | | | | | 0 | | | | 0 | | | | | 0 | | | | | 0 | | | | | 0 | | | |  |  |
| RUM | | WAG | | | 11 | | | | 0 | | | | | 0 | | | | | 1 | | | | | 1 | | | | | | | 0 | | | | 0 | | | | | 1 | | | | | 0 | | | | | 1 | | | |  |  |
| RUM | | WAG | | | 12 | | | | 1 | | | | | 1 | | | | | 3 | | | | | 0 | | | | | | | 2 | | | | 0 | | | | | 0 | | | | | 0 | | | | | 0 | | | |  |  |
| RUM | | WAG | | | 13 | | | | 0 | | | | | 0 | | | | | 2 | | | | | 0 | | | | | | | 0 | | | | 0 | | | | | 0 | | | | | 0 | | | | | 0 | | | |  |  |
| RUM | | WAG | | | 14 | | | | 1 | | | | | 0 | | | | | 2 | | | | | 0 | | | | | | | 0 | | | | 0 | | | | | 0 | | | | | 0 | | | | | 0 | | | |  |  |
| RUM | | CLY | | | 10 | | | | 0 | | | | | 0 | | | | | 0 | | | | | 0 | | | | | | | 0 | | | | 0 | | | | | 0 | | | | | 0 | | | | | 1 | | | |  |  |
| RUM | | CLY | | | 11 | | | | 0 | | | | | 0 | | | | | 0 | | | | | 0 | | | | | | | 0 | | | | 0 | | | | | 0 | | | | | 0 | | | | | 0 | | | |  |  |
| RUM | | CLY | | | 12 | | | | 0 | | | | | 0 | | | | | 0 | | | | | 1 | | | | | | | 0 | | | | 0 | | | | | 0 | | | | | 0 | | | | | 0 | | | |  |  |
| RUM | | CLY | | | 13 | | | | 0 | | | | | 0 | | | | | 0 | | | | | 0 | | | | | | | 0 | | | | 0 | | | | | 0 | | | | | 0 | | | | | 0 | | | |  |  |
| RUM | | CLY | | | 14 | | | | 0 | | | | | 0 | | | | | 1 | | | | | 0 | | | | | | | 0 | | | | 0 | | | | | 0 | | | | | 0 | | | | | 0 | | | |  |  |
| RUM | | CLY | | | 15 | | | | 0 | | | | | 1 | | | | | 1 | | | | | 0 | | | | | | | 0 | | | | 0 | | | | | 0 | | | | | 0 | | | | | 0 | | | |  |  |
| RUM | | CLY | | | 16 | | | | 0 | | | | | 0 | | | | | 1 | | | | | 0 | | | | | | | 0 | | | | 0 | | | | | 0 | | | | | 0 | | | | | 0 | | | |  |  |
| RUM | | JER | | | 9 | | | | 0 | | | | | 0 | | | | | 0 | | | | | 0 | | | | | | | 0 | | | | 0 | | | | | 0 | | | | | 0 | | | | | 0 | | | |  |  |
| RUM | | JER | | | 10 | | | | 0 | | | | | 0 | | | | | 0 | | | | | 0 | | | | | | | 0 | | | | 0 | | | | | 0 | | | | | 0 | | | | | 0 | | | |  |  |
| RUM | | JER | | | 11 | | | | 0 | | | | | 0 | | | | | 1 | | | | | 0 | | | | | | | 0 | | | | 0 | | | | | 0 | | | | | 0 | | | | | 0 | | | |  |  |
| RUM | | JER | | | 12 | | | | 0 | | | | | 0 | | | | | 0 | | | | | 0 | | | | | | | 0 | | | | 0 | | | | | 0 | | | | | 0 | | | | | 0 | | | |  |  |
| RUM | | JER | | | 13 | | | | 0 | | | | | 0 | | | | | 0 | | | | | 0 | | | | | | | 0 | | | | 0 | | | | | 0 | | | | | 0 | | | | | 0 | | | |  |  |
| RUM | | JER | | | 14 | | | | 0 | | | | | 0 | | | | | 0 | | | | | 0 | | | | | | | 0 | | | | 0 | | | | | 0 | | | | | 0 | | | | | 0 | | | |  |  |
| RUM | | JER | | | 15 | | | | 0 | | | | | 0 | | | | | 0 | | | | | 0 | | | | | | | 0 | | | | 0 | | | | | 0 | | | | | 0 | | | | | 0 | | | |  |  |
| **Mo** | | **Es** | | | **Si** | | | | **Goniadidae** | | | **Hesionidae** | | | | **Lumbrineridae** | | | | | | **Magelonidae** | | | | | **Maldonidae** | | | | | | **Nephtyidae** | | | | **Nereididae** | | | | | **Opheliidae** | | | | | | **Orbiniidae** | | | | | |  |  |
| RUM | | HAK | | | 15 | | | | 0 | | | 0 | | | | 1 | | | | | | 0 | | | | | 0 | | | | | | 0 | | | | 0 | | | | | 1 | | | | | | 1 | | | | | |  |  |
| RUM | | HAK | | | 17 | | | | 0 | | | 0 | | | | 0 | | | | | | 0 | | | | | 0 | | | | | | 0 | | | | 0 | | | | | 1 | | | | | | 0 | | | | | |  |  |
| RUM | | HAK | | | 19 | | | | 0 | | | 0 | | | | 0 | | | | | | 0 | | | | | 0 | | | | | | 0 | | | | 0 | | | | | 2 | | | | | | 0 | | | | | |  |  |
| RUM | | HAK | | | 20 | | | | 0 | | | 1 | | | | 0 | | | | | | 0 | | | | | 0 | | | | | | 0 | | | | 0 | | | | | 1 | | | | | | 0 | | | | | |  |  |
| RUM | | HAK | | | 25 | | | | 0 | | | 0 | | | | 0 | | | | | | 0 | | | | | 1 | | | | | | 1 | | | | 2 | | | | | 0 | | | | | | 0 | | | | | |  |  |
| RUM | | HAK | | | 26 | | | | 0 | | | 0 | | | | 0 | | | | | | 0 | | | | | 0 | | | | | | 0 | | | | 0 | | | | | 1 | | | | | | 0 | | | | | |  |  |
| RUM | | HAK | | | 27 | | | | 0 | | | 0 | | | | 0 | | | | | | 0 | | | | | 0 | | | | | | 0 | | | | 0 | | | | | 0 | | | | | | 0 | | | | | |  |  |
| RUM | | WAG | | | 8 | | | | 0 | | | 0 | | | | 0 | | | | | | 0 | | | | | 1 | | | | | | 0 | | | | 1 | | | | | 0 | | | | | | 0 | | | | | |  |  |
| RUM | | WAG | | | 9 | | | | 0 | | | 1 | | | | 1 | | | | | | 1 | | | | | 1 | | | | | | 0 | | | | 0 | | | | | 0 | | | | | | 0 | | | | | |  |  |
| RUM | | WAG | | | 10 | | | | 0 | | | 0 | | | | 0 | | | | | | 0 | | | | | 0 | | | | | | 0 | | | | 0 | | | | | 0 | | | | | | 0 | | | | | |  |  |
| RUM | | WAG | | | 11 | | | | 0 | | | 0 | | | | 1 | | | | | | 0 | | | | | 0 | | | | | | 1 | | | | 1 | | | | | 0 | | | | | | 0 | | | | | |  |  |
| RUM | | WAG | | | 12 | | | | 0 | | | 1 | | | | 1 | | | | | | 2 | | | | | 0 | | | | | | 1 | | | | 0 | | | | | 0 | | | | | | 0 | | | | | |  |  |
| RUM | | WAG | | | 13 | | | | 0 | | | 0 | | | | 1 | | | | | | 1 | | | | | 0 | | | | | | 1 | | | | 2 | | | | | 0 | | | | | | 0 | | | | | |  |  |
| RUM | | WAG | | | 14 | | | | 0 | | | 1 | | | | 1 | | | | | | 2 | | | | | 1 | | | | | | 0 | | | | 0 | | | | | 0 | | | | | | 0 | | | | | |  |  |
| RUM | | CLY | | | 10 | | | | 0 | | | 1 | | | | 0 | | | | | | 0 | | | | | 0 | | | | | | 0 | | | | 0 | | | | | 0 | | | | | | 3 | | | | | |  |  |
| RUM | | CLY | | | 11 | | | | 0 | | | 0 | | | | 0 | | | | | | 0 | | | | | 0 | | | | | | 0 | | | | 0 | | | | | 0 | | | | | | 3 | | | | | |  |  |
| RUM | | CLY | | | 12 | | | | 0 | | | 0 | | | | 0 | | | | | | 0 | | | | | 0 | | | | | | 0 | | | | 0 | | | | | 0 | | | | | | 0 | | | | | |  |  |
| RUM | | CLY | | | 13 | | | | 0 | | | 0 | | | | 0 | | | | | | 0 | | | | | 0 | | | | | | 0 | | | | 0 | | | | | 0 | | | | | | 0 | | | | | |  |  |
| RUM | | CLY | | | 14 | | | | 0 | | | 0 | | | | 0 | | | | | | 0 | | | | | 0 | | | | | | 0 | | | | 0 | | | | | 0 | | | | | | 0 | | | | | |  |  |
| RUM | | CLY | | | 15 | | | | 0 | | | 0 | | | | 0 | | | | | | 0 | | | | | 0 | | | | | | 0 | | | | 0 | | | | | 0 | | | | | | 1 | | | | | |  |  |
| RUM | | CLY | | | 16 | | | | 0 | | | 0 | | | | 0 | | | | | | 0 | | | | | 0 | | | | | | 0 | | | | 0 | | | | | 0 | | | | | | 0 | | | | | |  |  |
| RUM | | JER | | | 9 | | | | 0 | | | 1 | | | | 0 | | | | | | 0 | | | | | 0 | | | | | | 0 | | | | 0 | | | | | 0 | | | | | | 0 | | | | | |  |  |
| RUM | | JER | | | 10 | | | | 0 | | | 0 | | | | 0 | | | | | | 0 | | | | | 0 | | | | | | 0 | | | | 1 | | | | | 0 | | | | | | 0 | | | | | |  |  |
| RUM | | JER | | | 11 | | | | 0 | | | 2 | | | | 0 | | | | | | 0 | | | | | 0 | | | | | | 0 | | | | 0 | | | | | 0 | | | | | | 0 | | | | | |  |  |
| RUM | | JER | | | 12 | | | | 0 | | | 0 | | | | 0 | | | | | | 0 | | | | | 0 | | | | | | 1 | | | | 0 | | | | | 0 | | | | | | 1 | | | | | |  |  |
| RUM | | JER | | | 13 | | | | 0 | | | 1 | | | | 0 | | | | | | 0 | | | | | 0 | | | | | | 0 | | | | 0 | | | | | 0 | | | | | | 0 | | | | | |  |  |
| RUM | | JER | | | 14 | | | | 0 | | | 1 | | | | 0 | | | | | | 0 | | | | | 0 | | | | | | 0 | | | | 0 | | | | | 0 | | | | | | 0 | | | | | |  |  |
| RUM | | JER | | | 15 | | | | 0 | | | 2 | | | | 0 | | | | | | 0 | | | | | 0 | | | | | | 0 | | | | 0 | | | | | 0 | | | | | | 0 | | | | | |  |  |
| **Mo** | | **Es** | | | **Si** | | | | **Paraonidae** | | **Phyllodocidae** | | | | | | **Polynoidae** | | | | **Sabellidae** | | | | | **Scalibregmidae** | | | | | | **Sigalionidae** | | | | | | **Spionidae** | | | | | **Syllidae** | | | | **Terebellid** | | | | |  |  |  |  |
| RUM | | HAK | | | 15 | | | | 0 | | 1 | | | | | | 0 | | | | 0 | | | | | 0 | | | | | | 0 | | | | | | 1 | | | | | 0 | | | | 0 | | | | |  |  |  |  |
| RUM | | HAK | | | 17 | | | | 0 | | 1 | | | | | | 0 | | | | 0 | | | | | 0 | | | | | | 0 | | | | | | 2 | | | | | 0 | | | | 0 | | | | |  |  |  |  |
| RUM | | HAK | | | 19 | | | | 0 | | 0 | | | | | | 0 | | | | 0 | | | | | 0 | | | | | | 0 | | | | | | 2 | | | | | 3 | | | | 0 | | | | |  |  |  |  |
| RUM | | HAK | | | 20 | | | | 0 | | 1 | | | | | | 0 | | | | 0 | | | | | 0 | | | | | | 0 | | | | | | 2 | | | | | 2 | | | | 0 | | | | |  |  |  |  |
| RUM | | HAK | | | 25 | | | | 0 | | 2 | | | | | | 0 | | | | 0 | | | | | 0 | | | | | | 0 | | | | | | 7 | | | | | 3 | | | | 0 | | | | |  |  |  |  |
| RUM | | HAK | | | 26 | | | | 1 | | 0 | | | | | | 0 | | | | 0 | | | | | 0 | | | | | | 0 | | | | | | 1 | | | | | 1 | | | | 0 | | | | |  |  |  |  |
| RUM | | HAK | | | 27 | | | | 0 | | 0 | | | | | | 0 | | | | 0 | | | | | 0 | | | | | | 0 | | | | | | 3 | | | | | 2 | | | | 0 | | | | |  |  |  |  |
| RUM | | WAG | | | 8 | | | | 0 | | 0 | | | | | | 0 | | | | 0 | | | | | 0 | | | | | | 0 | | | | | | 0 | | | | | 3 | | | | 0 | | | | |  |  |  |  |
| RUM | | WAG | | | 9 | | | | 0 | | 0 | | | | | | 0 | | | | 0 | | | | | 0 | | | | | | 0 | | | | | | 2 | | | | | 3 | | | | 0 | | | | |  |  |  |  |
| RUM | | WAG | | | 10 | | | | 0 | | 0 | | | | | | 0 | | | | 0 | | | | | 0 | | | | | | 0 | | | | | | 4 | | | | | 1 | | | | 0 | | | | |  |  |  |  |
| RUM | | WAG | | | 11 | | | | 0 | | 2 | | | | | | 0 | | | | 0 | | | | | 0 | | | | | | 0 | | | | | | 1 | | | | | 7 | | | | 0 | | | | |  |  |  |  |
| RUM | | WAG | | | 12 | | | | 0 | | 0 | | | | | | 0 | | | | 0 | | | | | 0 | | | | | | 0 | | | | | | 1 | | | | | 8 | | | | 0 | | | | |  |  |  |  |
| RUM | | WAG | | | 13 | | | | 0 | | 1 | | | | | | 0 | | | | 0 | | | | | 1 | | | | | | 0 | | | | | | 3 | | | | | 8 | | | | 0 | | | | |  |  |  |  |
| RUM | | WAG | | | 14 | | | | 0 | | 0 | | | | | | 0 | | | | 0 | | | | | 0 | | | | | | 0 | | | | | | 2 | | | | | 2 | | | | 0 | | | | |  |  |  |  |
| RUM | | CLY | | | 10 | | | | 0 | | 0 | | | | | | 0 | | | | 0 | | | | | 0 | | | | | | 0 | | | | | | 1 | | | | | 0 | | | | 0 | | | | |  |  |  |  |
| RUM | | CLY | | | 11 | | | | 0 | | 0 | | | | | | 0 | | | | 0 | | | | | 0 | | | | | | 0 | | | | | | 2 | | | | | 0 | | | | 0 | | | | |  |  |  |  |
| RUM | | CLY | | | 12 | | | | 0 | | 0 | | | | | | 0 | | | | 0 | | | | | 0 | | | | | | 0 | | | | | | 0 | | | | | 1 | | | | 0 | | | | |  |  |  |  |
| RUM | | CLY | | | 13 | | | | 0 | | 0 | | | | | | 0 | | | | 0 | | | | | 0 | | | | | | 0 | | | | | | 0 | | | | | 0 | | | | 0 | | | | |  |  |  |  |
| RUM | | CLY | | | 14 | | | | 0 | | 0 | | | | | | 0 | | | | 0 | | | | | 0 | | | | | | 0 | | | | | | 3 | | | | | 0 | | | | 0 | | | | |  |  |  |  |
| RUM | | CLY | | | 15 | | | | 0 | | 0 | | | | | | 0 | | | | 0 | | | | | 0 | | | | | | 0 | | | | | | 3 | | | | | 0 | | | | 0 | | | | |  |  |  |  |
| RUM | | CLY | | | 16 | | | | 0 | | 0 | | | | | | 0 | | | | 0 | | | | | 0 | | | | | | 0 | | | | | | 0 | | | | | 0 | | | | 0 | | | | |  |  |  |  |
| RUM | | JER | | | 9 | | | | 0 | | 0 | | | | | | 0 | | | | 0 | | | | | 0 | | | | | | 0 | | | | | | 0 | | | | | 6 | | | | 0 | | | | |  |  |  |  |
| RUM | | JER | | | 10 | | | | 0 | | 0 | | | | | | 0 | | | | 0 | | | | | 0 | | | | | | 0 | | | | | | 2 | | | | | 3 | | | | 0 | | | | |  |  |  |  |
| RUM | | JER | | | 11 | | | | 0 | | 0 | | | | | | 0 | | | | 0 | | | | | 0 | | | | | | 0 | | | | | | 1 | | | | | 2 | | | | 0 | | | | |  |  |  |  |
| RUM | | JER | | | 12 | | | | 0 | | 0 | | | | | | 0 | | | | 0 | | | | | 0 | | | | | | 0 | | | | | | 1 | | | | | 2 | | | | 0 | | | | |  |  |  |  |
| RUM | | JER | | | 13 | | | | 0 | | 0 | | | | | | 0 | | | | 0 | | | | | 0 | | | | | | 0 | | | | | | 1 | | | | | 1 | | | | 0 | | | | |  |  |  |  |
| RUM | | JER | | | 14 | | | | 0 | | 0 | | | | | | 0 | | | | 0 | | | | | 0 | | | | | | 0 | | | | | | 1 | | | | | 2 | | | | 0 | | | | |  |  |  |  |
| RUM | | JER | | | 15 | | | | 0 | | 0 | | | | | | 0 | | | | 0 | | | | | 0 | | | | | | 0 | | | | | | 11 | | | | | 7 | | | | 0 | | | | |  |  |  |  |
| **Mo** | | **Es** | | | **Si** | | | | **Trichobranchidae** | | | | | | **Juveniles** | | | | | **Oligochaeta** | | | | | **Nematoda** | | | | | **Nemertea** | | | | **Sipuncula** | | | **Priapulida** | | | | | **Phoronida** | | | | | **Amphipoda** | | | | |  |  |  |  |
| RUM | | HAK | | | 15 | | | | 0 | | | | | | 0 | | | | | 0 | | | | | 2 | | | | | 2 | | | | 1 | | | 0 | | | | | 0 | | | | | 1 | | | | |  |  |  |  |
| RUM | | HAK | | | 17 | | | | 0 | | | | | | 0 | | | | | 0 | | | | | 40 | | | | | 4 | | | | 1 | | | 0 | | | | | 0 | | | | | 1 | | | | |  |  |  |  |
| RUM | | HAK | | | 19 | | | | 0 | | | | | | 0 | | | | | 1 | | | | | 2 | | | | | 1 | | | | 0 | | | 0 | | | | | 0 | | | | | 0 | | | | |  |  |  |  |
| RUM | | HAK | | | 20 | | | | 0 | | | | | | 0 | | | | | 0 | | | | | 38 | | | | | 7 | | | | 0 | | | 0 | | | | | 0 | | | | | 1 | | | | |  |  |  |  |
| RUM | | HAK | | | 25 | | | | 0 | | | | | | 0 | | | | | 4 | | | | | 25 | | | | | 9 | | | | 0 | | | 0 | | | | | 0 | | | | | 0 | | | | |  |  |  |  |
| RUM | | HAK | | | 26 | | | | 0 | | | | | | 0 | | | | | 15 | | | | | 10 | | | | | 2 | | | | 0 | | | 0 | | | | | 0 | | | | | 1 | | | | |  |  |  |  |
| RUM | | HAK | | | 27 | | | | 0 | | | | | | 0 | | | | | 0 | | | | | 23 | | | | | 4 | | | | 0 | | | 0 | | | | | 0 | | | | | 1 | | | | |  |  |  |  |
| RUM | | WAG | | | 8 | | | | 0 | | | | | | 0 | | | | | 3 | | | | | 31 | | | | | 3 | | | | 1 | | | 0 | | | | | 0 | | | | | 0 | | | | |  |  |  |  |
| RUM | | WAG | | | 9 | | | | 0 | | | | | | 0 | | | | | 0 | | | | | 51 | | | | | 3 | | | | 1 | | | 0 | | | | | 0 | | | | | 0 | | | | |  |  |  |  |
| RUM | | WAG | | | 10 | | | | 0 | | | | | | 0 | | | | | 1 | | | | | 45 | | | | | 3 | | | | 0 | | | 1 | | | | | 0 | | | | | 1 | | | | |  |  |  |  |
| RUM | | WAG | | | 11 | | | | 0 | | | | | | 0 | | | | | 0 | | | | | 60 | | | | | 3 | | | | 1 | | | 0 | | | | | 0 | | | | | 0 | | | | |  |  |  |  |
| RUM | | WAG | | | 12 | | | | 0 | | | | | | 1 | | | | | 0 | | | | | 26 | | | | | 6 | | | | 4 | | | 0 | | | | | 0 | | | | | 0 | | | | |  |  |  |  |
| RUM | | WAG | | | 13 | | | | 0 | | | | | | 0 | | | | | 0 | | | | | 89 | | | | | 1 | | | | 0 | | | 0 | | | | | 0 | | | | | 3 | | | | |  |  |  |  |
| RUM | | WAG | | | 14 | | | | 0 | | | | | | 0 | | | | | 0 | | | | | 19 | | | | | 1 | | | | 0 | | | 0 | | | | | 0 | | | | | 0 | | | | |  |  |  |  |
| RUM | | CLY | | | 10 | | | | 0 | | | | | | 0 | | | | | 0 | | | | | 1 | | | | | 1 | | | | 0 | | | 0 | | | | | 0 | | | | | 0 | | | | |  |  |  |  |
| RUM | | CLY | | | 11 | | | | 0 | | | | | | 0 | | | | | 0 | | | | | 1 | | | | | 1 | | | | 0 | | | 0 | | | | | 0 | | | | | 0 | | | | |  |  |  |  |
| RUM | | CLY | | | 12 | | | | 0 | | | | | | 0 | | | | | 0 | | | | | 3 | | | | | 1 | | | | 0 | | | 0 | | | | | 0 | | | | | 0 | | | | |  |  |  |  |
| RUM | | CLY | | | 13 | | | | 0 | | | | | | 0 | | | | | 0 | | | | | 0 | | | | | 0 | | | | 0 | | | 0 | | | | | 0 | | | | | 0 | | | | |  |  |  |  |
| RUM | | CLY | | | 14 | | | | 0 | | | | | | 0 | | | | | 0 | | | | | 1 | | | | | 0 | | | | 0 | | | 0 | | | | | 0 | | | | | 0 | | | | |  |  |  |  |
| RUM | | CLY | | | 15 | | | | 0 | | | | | | 0 | | | | | 0 | | | | | 1 | | | | | 0 | | | | 0 | | | 0 | | | | | 0 | | | | | 0 | | | | |  |  |  |  |
| RUM | | CLY | | | 16 | | | | 0 | | | | | | 0 | | | | | 0 | | | | | 0 | | | | | 0 | | | | 0 | | | 0 | | | | | 0 | | | | | 0 | | | | |  |  |  |  |
| RUM | | JER | | | 9 | | | | 0 | | | | | | 0 | | | | | 0 | | | | | 19 | | | | | 2 | | | | 0 | | | 0 | | | | | 0 | | | | | 2 | | | | |  |  |  |  |
| RUM | | JER | | | 10 | | | | 0 | | | | | | 0 | | | | | 0 | | | | | 4 | | | | | 0 | | | | 0 | | | 0 | | | | | 0 | | | | | 1 | | | | |  |  |  |  |
| RUM | | JER | | | 11 | | | | 0 | | | | | | 0 | | | | | 3 | | | | | 2 | | | | | 1 | | | | 0 | | | 0 | | | | | 0 | | | | | 1 | | | | |  |  |  |  |
| RUM | | JER | | | 12 | | | | 0 | | | | | | 0 | | | | | 0 | | | | | 0 | | | | | 0 | | | | 0 | | | 0 | | | | | 0 | | | | | 1 | | | | |  |  |  |  |
| RUM | | JER | | | 13 | | | | 0 | | | | | | 0 | | | | | 0 | | | | | 4 | | | | | 0 | | | | 0 | | | 0 | | | | | 0 | | | | | 0 | | | | |  |  |  |  |
| RUM | | JER | | | 14 | | | | 0 | | | | | | 0 | | | | | 0 | | | | | 8 | | | | | 2 | | | | 0 | | | 0 | | | | | 0 | | | | | 1 | | | | |  |  |  |  |
| RUM | | JER | | | 15 | | | | 0 | | | | | | 0 | | | | | 1 | | | | | 2 | | | | | 0 | | | | 0 | | | 0 | | | | | 0 | | | | | 1 | | | | |  |  |  |  |
| **Mo** | | | | **Es** | | | **Si** | **Copepoda** | | | **Isopoda** | | | | | **Ostrocoda** | | | | | **Tanaidacea** | | | | | **Cumacea** | | | **Decapoda** | | | **Brachyura** | | | | **Bivalvia** | | | | | **Gastropoda** | | | | | **Echinoidea** | | | | | **Asteroidea** | | | | |
| RUM | | | | HAK | | | 15 | 0 | | | 1 | | | | | 5 | | | | | 0 | | | | | 0 | | | 0 | | | 1 | | | | 0 | | | | | 0 | | | | | 0 | | | | | 0 | | | | |
| RUM | | | | HAK | | | 17 | 0 | | | 1 | | | | | 1 | | | | | 0 | | | | | 0 | | | 0 | | | 0 | | | | 1 | | | | | 0 | | | | | 0 | | | | | 0 | | | | |
| RUM | | | | HAK | | | 19 | 1 | | | 1 | | | | | 3 | | | | | 0 | | | | | 0 | | | 0 | | | 0 | | | | 2 | | | | | 2 | | | | | 0 | | | | | 1 | | | | |
| RUM | | | | HAK | | | 20 | 3 | | | 1 | | | | | 3 | | | | | 0 | | | | | 1 | | | 0 | | | 0 | | | | 4 | | | | | 0 | | | | | 0 | | | | | 1 | | | | |
| RUM | | | | HAK | | | 25 | 3 | | | 2 | | | | | 4 | | | | | 2 | | | | | 1 | | | 1 | | | 0 | | | | 1 | | | | | 1 | | | | | 1 | | | | | 0 | | | | |
| RUM | | | | HAK | | | 26 | 0 | | | 1 | | | | | 1 | | | | | 0 | | | | | 0 | | | 1 | | | 0 | | | | 1 | | | | | 0 | | | | | 0 | | | | | 0 | | | | |
| RUM | | | | HAK | | | 27 | 7 | | | 1 | | | | | 0 | | | | | 0 | | | | | 0 | | | 0 | | | 0 | | | | 0 | | | | | 0 | | | | | 0 | | | | | 0 | | | | |
| RUM | | | | WAG | | | 8 | 0 | | | 0 | | | | | 0 | | | | | 0 | | | | | 0 | | | 0 | | | 0 | | | | 0 | | | | | 0 | | | | | 0 | | | | | 0 | | | | |
| RUM | | | | WAG | | | 9 | 2 | | | 0 | | | | | 0 | | | | | 0 | | | | | 0 | | | 0 | | | 0 | | | | 1 | | | | | 0 | | | | | 0 | | | | | 0 | | | | |
| RUM | | | | WAG | | | 10 | 4 | | | 0 | | | | | 2 | | | | | 0 | | | | | 0 | | | 0 | | | 0 | | | | 0 | | | | | 0 | | | | | 0 | | | | | 0 | | | | |
| RUM | | | | WAG | | | 11 | 2 | | | 1 | | | | | 0 | | | | | 0 | | | | | 3 | | | 0 | | | 0 | | | | 1 | | | | | 0 | | | | | 0 | | | | | 0 | | | | |
| RUM | | | | WAG | | | 12 | 1 | | | 0 | | | | | 0 | | | | | 1 | | | | | 0 | | | 1 | | | 0 | | | | 1 | | | | | 0 | | | | | 1 | | | | | 0 | | | | |
| RUM | | | | WAG | | | 13 | 10 | | | 0 | | | | | 1 | | | | | 0 | | | | | 1 | | | 0 | | | 0 | | | | 1 | | | | | 0 | | | | | 0 | | | | | 0 | | | | |
| RUM | | | | WAG | | | 14 | 1 | | | 0 | | | | | 0 | | | | | 0 | | | | | 0 | | | 0 | | | 0 | | | | 1 | | | | | 0 | | | | | 0 | | | | | 0 | | | | |
| RUM | | | | CLY | | | 10 | 1 | | | 0 | | | | | 0 | | | | | 0 | | | | | 1 | | | 0 | | | 0 | | | | 1 | | | | | 0 | | | | | 0 | | | | | 0 | | | | |
| RUM | | | | CLY | | | 11 | 0 | | | 0 | | | | | 0 | | | | | 0 | | | | | 0 | | | 0 | | | 0 | | | | 1 | | | | | 0 | | | | | 0 | | | | | 0 | | | | |
| RUM | | | | CLY | | | 12 | 1 | | | 0 | | | | | 0 | | | | | 0 | | | | | 0 | | | 0 | | | 0 | | | | 0 | | | | | 0 | | | | | 0 | | | | | 0 | | | | |
| RUM | | | | CLY | | | 13 | 0 | | | 0 | | | | | 0 | | | | | 0 | | | | | 0 | | | 0 | | | 0 | | | | 0 | | | | | 0 | | | | | 0 | | | | | 0 | | | | |
| RUM | | | | CLY | | | 14 | 0 | | | 0 | | | | | 0 | | | | | 0 | | | | | 0 | | | 0 | | | 0 | | | | 0 | | | | | 0 | | | | | 0 | | | | | 0 | | | | |
| RUM | | | | CLY | | | 15 | 0 | | | 0 | | | | | 0 | | | | | 0 | | | | | 0 | | | 0 | | | 0 | | | | 0 | | | | | 0 | | | | | 0 | | | | | 0 | | | | |
| RUM | | | | CLY | | | 16 | 0 | | | 0 | | | | | 0 | | | | | 0 | | | | | 0 | | | 0 | | | 0 | | | | 2 | | | | | 0 | | | | | 0 | | | | | 0 | | | | |
| RUM | | | | JER | | | 9 | 1 | | | 0 | | | | | 1 | | | | | 0 | | | | | 2 | | | 0 | | | 0 | | | | 0 | | | | | 0 | | | | | 0 | | | | | 0 | | | | |
| RUM | | | | JER | | | 10 | 0 | | | 0 | | | | | 0 | | | | | 0 | | | | | 0 | | | 0 | | | 0 | | | | 1 | | | | | 0 | | | | | 0 | | | | | 0 | | | | |
| RUM | | | | JER | | | 11 | 1 | | | 0 | | | | | 0 | | | | | 0 | | | | | 0 | | | 1 | | | 0 | | | | 0 | | | | | 0 | | | | | 0 | | | | | 0 | | | | |
| RUM | | | | JER | | | 12 | 0 | | | 0 | | | | | 2 | | | | | 0 | | | | | 0 | | | 0 | | | 0 | | | | 1 | | | | | 0 | | | | | 0 | | | | | 0 | | | | |
| RUM | | | | JER | | | 13 | 0 | | | 0 | | | | | 1 | | | | | 0 | | | | | 1 | | | 0 | | | 0 | | | | 0 | | | | | 0 | | | | | 0 | | | | | 0 | | | | |
| RUM | | | | JER | | | 14 | 0 | | | 0 | | | | | 0 | | | | | 0 | | | | | 1 | | | 0 | | | 0 | | | | 0 | | | | | 1 | | | | | 0 | | | | | 0 | | | | |
| RUM | | | | JER | | | 15 | 0 | | | 0 | | | | | 0 | | | | | 0 | | | | | 0 | | | 0 | | | 0 | | | | 0 | | | | | 0 | | | | | 0 | | | | | 0 | | | | |
