# Supplementary material for: Polychaete Richness and Abundance Enhanced in Anthropogenically Modified Estuaries Despite High Concentrations of Toxic Contaminants
Source: PLoS One. 2013 Sep 30;8(9):e77018. doi: 10.1371/journal.pone.0077018 (PMC3786951; doi:10.1371/journal.pone.0077018)
Supplement: Table S3 — Soft sediment infauna sampled in field manipulated sediment. (DOCX) [file pone.0077018.s007.docx]

**Table S3.** Soft sediment infauna sampled in field manipulated sediment. Sediment was sourced from six NSW estuaries that were either heavily modified or relatively unmodified and deployed in benthic recruitment containers in the Clyde. Modification category (Mo; heavily modified (HM) or relatively unmodified (RUM)) and Estuary (Es; nested in Modification category). BRCs were the replicates.

| **Mo** | **Es** | **Rep** | ***Barantolla lepta*** | ***Capitella* sp.** | ***Heteromastus filiformis*** | ***Mediomastus australiensis*** | ***Notomastus chrysosetus*** | ***Notomastus* sp.** |
| --- | --- | --- | --- | --- | --- | --- | --- | --- |
| HM | KEM | 1 | 1 | 1 | 0 | 0 | 0 | 0 |
| HM | KEM | 2 | 0 | 4 | 0 | 0 | 0 | 0 |
| HM | KEM | 3 | 0 | 0 | 0 | 0 | 0 | 0 |
| HM | KEM | 4 | 0 | 0 | 0 | 1 | 0 | 0 |
| HM | KEM | 5 | 0 | 7 | 0 | 0 | 0 | 0 |
| HM | KEM | 6 | 0 | 0 | 0 | 0 | 0 | 0 |
| HM | KEM | 7 | 3 | 0 | 0 | 0 | 0 | 0 |
| HM | JAK | 1 | 0 | 0 | 0 | 0 | 0 | 0 |
| HM | JAK | 2 | 0 | 2 | 0 | 0 | 0 | 0 |
| HM | JAK | 4 | 0 | 4 | 0 | 1 | 0 | 0 |
| HM | JAK | 5 | 0 | 0 | 0 | 1 | 0 | 0 |
| HM | JAK | 6 | 0 | 66 | 0 | 0 | 0 | 0 |
| HM | JAK | 7 | 0 | 0 | 0 | 0 | 0 | 0 |
| HM | BOT | 1 | 0 | 0 | 0 | 1 | 0 | 0 |
| HM | BOT | 2 | 0 | 0 | 0 | 2 | 0 | 0 |
| HM | BOT | 3 | 0 | 0 | 0 | 0 | 0 | 1 |
| HM | BOT | 4 | 6 | 0 | 0 | 1 | 0 | 0 |
| HM | BOT | 5 | 0 | 2 | 1 | 2 | 0 | 0 |
| HM | BOT | 6 | 0 | 0 | 0 | 1 | 0 | 0 |
| HM | BOT | 7 | 0 | 0 | 0 | 0 | 0 | 0 |

| **Mo** | **Es** | **Rep** | ***Scyphoproctus* sp.** | ***Cossura* sp.** | ***Nematonereis* sp.** | ***Glycera tridactyla*** | ***Magelonis* sp.** | ***Nephtys australiensis*** | ***Nephtys inornata*** |
| --- | --- | --- | --- | --- | --- | --- | --- | --- | --- |
| HM | KEM | 1 | 0 | 0 | 0 | 0 | 0 | 0 | 1 |
| HM | KEM | 2 | 0 | 3 | 0 | 0 | 0 | 0 | 0 |
| HM | KEM | 3 | 0 | 0 | 0 | 0 | 0 | 1 | 0 |
| HM | KEM | 4 | 0 | 1 | 0 | 0 | 0 | 0 | 0 |
| HM | KEM | 5 | 0 | 0 | 0 | 0 | 0 | 0 | 0 |
| HM | KEM | 6 | 0 | 0 | 0 | 1 | 0 | 0 | 2 |
| HM | KEM | 7 | 0 | 1 | 0 | 0 | 0 | 0 | 0 |
| HM | JAK | 1 | 0 | 1 | 0 | 0 | 0 | 0 | 0 |
| HM | JAK | 2 | 0 | 2 | 0 | 0 | 0 | 0 | 0 |
| HM | JAK | 4 | 0 | 10 | 0 | 0 | 0 | 0 | 0 |
| HM | JAK | 5 | 0 | 3 | 0 | 1 | 0 | 0 | 0 |
| HM | JAK | 6 | 0 | 0 | 0 | 0 | 0 | 0 | 1 |
| HM | JAK | 7 | 0 | 0 | 0 | 1 | 0 | 0 | 0 |
| HM | BOT | 1 | 0 | 0 | 0 | 1 | 0 | 0 | 2 |
| HM | BOT | 2 | 0 | 6 | 0 | 1 | 0 | 0 | 0 |
| HM | BOT | 3 | 0 | 1 | 1 | 0 | 0 | 0 | 0 |
| HM | BOT | 4 | 0 | 8 | 0 | 0 | 0 | 0 | 0 |
| HM | BOT | 5 | 0 | 2 | 0 | 1 | 0 | 0 | 0 |
| HM | BOT | 6 | 0 | 0 | 0 | 0 | 0 | 0 | 0 |
| HM | BOT | 7 | 0 | 1 | 0 | 0 | 0 | 0 | 0 |

| **Mo** | **Es** | **Rep** | **Nereid sp.1** | ***Oenone* sp.** | ***Armandia intermedia*** | ***Scoloplos (Leodomos) johnstoni*** | ***Scoloplos (Scoloplos) simplex*** | ***Aricidea* sp.** |
| --- | --- | --- | --- | --- | --- | --- | --- | --- |
| HM | KEM | 1 | 0 | 0 | 0 | 0 | 0 | 0 |
| HM | KEM | 2 | 0 | 0 | 0 | 0 | 0 | 0 |
| HM | KEM | 3 | 0 | 1 | 0 | 0 | 0 | 0 |
| HM | KEM | 4 | 0 | 0 | 0 | 0 | 1 | 0 |
| HM | KEM | 5 | 0 | 0 | 0 | 0 | 0 | 0 |
| HM | KEM | 6 | 0 | 0 | 0 | 0 | 0 | 0 |
| HM | KEM | 7 | 0 | 0 | 0 | 0 | 0 | 1 |
| HM | JAK | 1 | 0 | 0 | 0 | 0 | 0 | 0 |
| HM | JAK | 2 | 0 | 0 | 0 | 0 | 0 | 0 |
| HM | JAK | 4 | 0 | 0 | 0 | 0 | 0 | 0 |
| HM | JAK | 5 | 0 | 0 | 0 | 0 | 2 | 0 |
| HM | JAK | 6 | 0 | 0 | 0 | 0 | 0 | 0 |
| HM | JAK | 7 | 0 | 0 | 0 | 0 | 3 | 0 |
| HM | BOT | 1 | 0 | 0 | 0 | 0 | 0 | 1 |
| HM | BOT | 2 | 0 | 0 | 0 | 0 | 0 | 0 |
| HM | BOT | 3 | 1 | 0 | 0 | 0 | 0 | 0 |
| HM | BOT | 4 | 0 | 0 | 0 | 0 | 0 | 2 |
| HM | BOT | 5 | 0 | 0 | 0 | 0 | 0 | 1 |
| HM | BOT | 6 | 0 | 0 | 0 | 0 | 0 | 0 |
| HM | BOT | 7 | 0 | 0 | 0 | 0 | 0 | 0 |

| **Mo** | **Es** | **Rep** | ***Paraonis* sp.** | ***Pectinaria australis*** | ***Paralepidonotus ampulliferus*** | ***Euchone variabilis*** | ***Prionospio* *wambiri*** | ***Prionospio tatura*** |
| --- | --- | --- | --- | --- | --- | --- | --- | --- |
| HM | KEM | 1 | 1 | 0 | 2 | 1 | 0 | 0 |
| HM | KEM | 2 | 0 | 0 | 0 | 0 | 0 | 4 |
| HM | KEM | 3 | 0 | 0 | 0 | 0 | 0 | 0 |
| HM | KEM | 4 | 2 | 0 | 1 | 0 | 0 | 2 |
| HM | KEM | 5 | 0 | 0 | 0 | 0 | 0 | 1 |
| HM | KEM | 6 | 0 | 0 | 1 | 0 | 0 | 0 |
| HM | KEM | 7 | 3 | 0 | 0 | 0 | 1 | 1 |
| HM | JAK | 1 | 1 | 0 | 1 | 0 | 2 | 1 |
| HM | JAK | 2 | 0 | 0 | 0 | 0 | 1 | 0 |
| HM | JAK | 4 | 2 | 0 | 0 | 0 | 2 | 0 |
| HM | JAK | 5 | 0 | 0 | 0 | 0 | 0 | 0 |
| HM | JAK | 6 | 0 | 0 | 1 | 0 | 0 | 3 |
| HM | JAK | 7 | 0 | 0 | 0 | 0 | 2 | 2 |
| HM | BOT | 1 | 0 | 0 | 0 | 0 | 0 | 0 |
| HM | BOT | 2 | 0 | 0 | 0 | 0 | 6 | 0 |
| HM | BOT | 3 | 1 | 0 | 1 | 0 | 3 | 0 |
| HM | BOT | 4 | 0 | 0 | 0 | 0 | 0 | 6 |
| HM | BOT | 5 | 0 | 0 | 0 | 0 | 0 | 3 |
| HM | BOT | 6 | 0 | 0 | 0 | 0 | 0 | 0 |
| HM | BOT | 7 | 0 | 0 | 0 | 0 | 0 | 0 |

| **Mo** | **Es** | **Rep** | ***Paraprionospio* sp.** | ***Prionospio yuriel*** | ***Pseudopolydora* sp*.*** | ***Carraziella* sp.** | ***Polydora* sp.** | **Exogene sp.1** | **Exogene sp. 2** |
| --- | --- | --- | --- | --- | --- | --- | --- | --- | --- |
| HM | KEM | 1 | 2 | 0 | 1 | 0 | 0 | 0 | 0 |
| HM | KEM | 2 | 0 | 0 | 1 | 0 | 0 | 0 | 0 |
| HM | KEM | 3 | 0 | 0 | 1 | 0 | 0 | 0 | 0 |
| HM | KEM | 4 | 1 | 0 | 4 | 0 | 0 | 0 | 0 |
| HM | KEM | 5 | 0 | 0 | 2 | 0 | 0 | 0 | 0 |
| HM | KEM | 6 | 1 | 0 | 1 | 0 | 0 | 1 | 0 |
| HM | KEM | 7 | 6 | 0 | 1 | 2 | 0 | 2 | 0 |
| HM | JAK | 1 | 2 | 0 | 4 | 0 | 0 | 0 | 0 |
| HM | JAK | 2 | 0 | 1 | 0 | 0 | 0 | 1 | 0 |
| HM | JAK | 4 | 0 | 0 | 3 | 0 | 0 | 0 | 0 |
| HM | JAK | 5 | 1 | 1 | 0 | 0 | 0 | 1 | 0 |
| HM | JAK | 6 | 0 | 0 | 0 | 1 | 0 | 2 | 0 |
| HM | JAK | 7 | 1 | 0 | 0 | 0 | 0 | 1 | 0 |
| HM | BOT | 1 | 0 | 0 | 0 | 0 | 0 | 0 | 0 |
| HM | BOT | 2 | 0 | 2 | 1 | 0 | 0 | 0 | 0 |
| HM | BOT | 3 | 0 | 0 | 0 | 0 | 1 | 1 | 0 |
| HM | BOT | 4 | 2 | 0 | 0 | 0 | 0 | 1 | 0 |
| HM | BOT | 5 | 0 | 0 | 1 | 0 | 0 | 0 | 0 |
| HM | BOT | 6 | 1 | 1 | 1 | 0 | 0 | 0 | 0 |
| HM | BOT | 7 | 0 | 0 | 1 | 0 | 0 | 0 | 0 |

| **Mo** | **Es** | **Rep** | **Exogene sp.3** | ***Astreptosyllis similiseta*** | **Syllinae sp.1** | **Syllinae sp.2** | ***Syllis* cf. *chrysillus*** | **Syllid/Phyllodocidae** | ***Lysilla* sp.** |
| --- | --- | --- | --- | --- | --- | --- | --- | --- | --- |
| HM | KEM | 1 | 0 | 0 | 0 | 0 | 0 | 0 | 0 |
| HM | KEM | 2 | 0 | 0 | 0 | 0 | 0 | 0 | 0 |
| HM | KEM | 3 | 0 | 0 | 0 | 0 | 0 | 0 | 0 |
| HM | KEM | 4 | 0 | 0 | 0 | 0 | 0 | 0 | 1 |
| HM | KEM | 5 | 0 | 0 | 0 | 0 | 0 | 0 | 0 |
| HM | KEM | 6 | 0 | 0 | 0 | 0 | 0 | 0 | 0 |
| HM | KEM | 7 | 0 | 0 | 0 | 0 | 0 | 0 | 0 |
| HM | JAK | 1 | 0 | 0 | 1 | 0 | 0 | 0 | 0 |
| HM | JAK | 2 | 0 | 0 | 0 | 0 | 0 | 0 | 0 |
| HM | JAK | 4 | 0 | 0 | 0 | 0 | 0 | 0 | 0 |
| HM | JAK | 5 | 0 | 0 | 0 | 0 | 0 | 0 | 0 |
| HM | JAK | 6 | 0 | 0 | 0 | 0 | 0 | 0 | 0 |
| HM | JAK | 7 | 0 | 0 | 0 | 0 | 0 | 0 | 0 |
| HM | BOT | 1 | 0 | 0 | 0 | 1 | 0 | 0 | 0 |
| HM | BOT | 2 | 0 | 0 | 1 | 0 | 0 | 0 | 0 |
| HM | BOT | 3 | 0 | 1 | 0 | 0 | 0 | 0 | 1 |
| HM | BOT | 4 | 0 | 0 | 0 | 0 | 0 | 0 | 0 |
| HM | BOT | 5 | 0 | 0 | 0 | 0 | 0 | 0 | 0 |
| HM | BOT | 6 | 2 | 0 | 0 | 0 | 0 | 0 | 0 |
| HM | BOT | 7 | 0 | 0 | 0 | 1 | 0 | 1 | 0 |

| **Mo** | **Es** | **Rep** | ***Ameania* sp.** | **Oligochaete** | **Nematode** | **Nemertean** | **Sipuncula** | **Amphipoda** | **Copepoda** | **Decapoda** | **Isopoda** | **Tanaidacea** | **Cumacea** |
| --- | --- | --- | --- | --- | --- | --- | --- | --- | --- | --- | --- | --- | --- |
| HM | KEM | 1 | 0 | 0 | 0 | 2 | 0 | 4 | 0 | 0 | 0 | 0 | 0 |
| HM | KEM | 2 | 0 | 0 | 0 | 1 | 0 | 18 | 0 | 0 | 0 | 1 | 0 |
| HM | KEM | 3 | 0 | 0 | 0 | 1 | 0 | 3 | 0 | 0 | 0 | 0 | 0 |
| HM | KEM | 4 | 1 | 0 | 0 | 1 | 0 | 6 | 0 | 0 | 0 | 0 | 0 |
| HM | KEM | 5 | 0 | 0 | 0 | 1 | 0 | 0 | 0 | 0 | 0 | 0 | 0 |
| HM | KEM | 6 | 0 | 0 | 0 | 0 | 0 | 6 | 0 | 0 | 0 | 0 | 0 |
| HM | KEM | 7 | 0 | 0 | 0 | 1 | 0 | 2 | 0 | 0 | 0 | 0 | 0 |
| HM | JAK | 1 | 0 | 0 | 0 | 1 | 1 | 4 | 0 | 1 | 0 | 0 | 0 |
| HM | JAK | 2 | 0 | 1 | 0 | 0 | 0 | 2 | 0 | 1 | 0 | 1 | 0 |
| HM | JAK | 4 | 0 | 0 | 0 | 0 | 0 | 3 | 0 | 0 | 0 | 0 | 0 |
| HM | JAK | 5 | 0 | 0 | 0 | 0 | 0 | 1 | 0 | 0 | 0 | 0 | 0 |
| HM | JAK | 6 | 0 | 0 | 0 | 0 | 0 | 2 | 0 | 0 | 0 | 0 | 0 |
| HM | JAK | 7 | 0 | 0 | 0 | 1 | 0 | 6 | 0 | 0 | 0 | 0 | 0 |
| HM | BOT | 1 | 0 | 0 | 0 | 0 | 0 | 6 | 0 | 0 | 0 | 0 | 0 |
| HM | BOT | 2 | 0 | 0 | 0 | 0 | 0 | 6 | 0 | 1 | 0 | 1 | 0 |
| HM | BOT | 3 | 0 | 0 | 0 | 0 | 0 | 9 | 0 | 1 | 0 | 0 | 0 |
| HM | BOT | 4 | 0 | 0 | 0 | 3 | 1 | 9 | 0 | 0 | 0 | 1 | 0 |
| HM | BOT | 5 | 0 | 0 | 1 | 0 | 0 | 0 | 0 | 0 | 0 | 0 | 0 |
| HM | BOT | 6 | 0 | 0 | 0 | 2 | 0 | 3 | 0 | 0 | 0 | 1 | 0 |
| HM | BOT | 7 | 0 | 0 | 0 | 2 | 0 | 1 | 0 | 0 | 0 | 0 | 0 |

| **Mo** | **Es** | **Rep** | **Gastropoda** | **Juveniles** |
| --- | --- | --- | --- | --- |
| HM | KEM | 1 | 0 | 0 |
| HM | KEM | 2 | 0 | 2 |
| HM | KEM | 3 | 0 | 0 |
| HM | KEM | 4 | 0 | 0 |
| HM | KEM | 5 | 0 | 0 |
| HM | KEM | 6 | 0 | 0 |
| HM | KEM | 7 | 0 | 0 |
| HM | JAK | 1 | 0 | 0 |
| HM | JAK | 2 | 0 | 1 |
| HM | JAK | 4 | 0 | 0 |
| HM | JAK | 5 | 0 | 0 |
| HM | JAK | 6 | 0 | 0 |
| HM | JAK | 7 | 0 | 0 |
| HM | BOT | 1 | 0 | 0 |
| HM | BOT | 2 | 0 | 0 |
| HM | BOT | 3 | 0 | 0 |
| HM | BOT | 4 | 0 | 0 |
| HM | BOT | 5 | 0 | 0 |
| HM | BOT | 6 | 0 | 0 |
| HM | BOT | 7 | 0 | 0 |

| **Mo** | **Es** | **Rep** | ***Barantolla lepta*** | ***Capitella* sp.** | ***Heteromastus filiformis*** | ***Mediomastus australiensis*** | ***Notomastus chrysosetus*** | ***Notomastus* sp.** |
| --- | --- | --- | --- | --- | --- | --- | --- | --- |
| RUM | HAK | 1 | 0 | 0 | 0 | 0 | 0 | 1 |
| RUM | HAK | 2 | 0 | 19 | 0 | 1 | 0 | 0 |
| RUM | HAK | 3 | 0 | 0 | 0 | 1 | 0 | 0 |
| RUM | HAK | 4 | 0 | 0 | 0 | 1 | 1 | 0 |
| RUM | HAK | 6 | 0 | 13 | 0 | 0 | 0 | 0 |
| RUM | HAK | 7 | 0 | 3 | 0 | 1 | 0 | 0 |
| RUM | WAG | 1 | 0 | 0 | 0 | 0 | 0 | 0 |
| RUM | WAG | 2 | 0 | 0 | 1 | 0 | 0 | 0 |
| RUM | WAG | 3 | 0 | 0 | 0 | 0 | 0 | 0 |
| RUM | WAG | 4 | 0 | 0 | 1 | 0 | 0 | 0 |
| RUM | WAG | 5 | 0 | 0 | 1 | 0 | 0 | 0 |
| RUM | WAG | 6 | 0 | 0 | 3 | 0 | 0 | 0 |
| RUM | WAG | 7 | 0 | 0 | 2 | 0 | 0 | 0 |
| RUM | CLY | 1 | 0 | 0 | 0 | 0 | 0 | 0 |
| RUM | CLY | 2 | 0 | 0 | 0 | 0 | 0 | 0 |
| RUM | CLY | 3 | 0 | 0 | 0 | 0 | 0 | 0 |
| RUM | CLY | 4 | 0 | 0 | 0 | 0 | 0 | 0 |
| RUM | CLY | 5 | 0 | 0 | 0 | 1 | 0 | 0 |
| RUM | CLY | 6 | 2 | 0 | 0 | 0 | 0 | 1 |
| RUM | CLY | 7 | 0 | 0 | 0 | 2 | 0 | 0 |

| **Mo** | **Es** | **Rep** | ***Scyphoproctus* sp.** | ***Cossura* sp.** | ***Nematonereis* sp.** | ***Glycera tridactyla*** | ***Magelonis* sp.** | ***Nephtys australiensis*** | ***Nephtys inornata*** |
| --- | --- | --- | --- | --- | --- | --- | --- | --- | --- |
| RUM | HAK | 1 | 0 | 0 | 0 | 0 | 0 | 0 | 1 |
| RUM | HAK | 2 | 0 | 0 | 0 | 0 | 1 | 2 | 0 |
| RUM | HAK | 3 | 0 | 1 | 0 | 0 | 0 | 0 | 0 |
| RUM | HAK | 4 | 0 | 0 | 0 | 0 | 0 | 0 | 0 |
| RUM | HAK | 6 | 0 | 2 | 0 | 0 | 0 | 0 | 0 |
| RUM | HAK | 7 | 0 | 0 | 0 | 0 | 0 | 0 | 0 |
| RUM | WAG | 1 | 0 | 0 | 0 | 0 | 0 | 1 | 0 |
| RUM | WAG | 2 | 0 | 7 | 0 | 0 | 0 | 0 | 0 |
| RUM | WAG | 3 | 0 | 1 | 0 | 0 | 0 | 0 | 0 |
| RUM | WAG | 4 | 1 | 11 | 0 | 0 | 0 | 0 | 0 |
| RUM | WAG | 5 | 0 | 1 | 0 | 0 | 0 | 1 | 0 |
| RUM | WAG | 6 | 0 | 4 | 0 | 0 | 1 | 0 | 0 |
| RUM | WAG | 7 | 0 | 4 | 0 | 1 | 0 | 0 | 0 |
| RUM | CLY | 1 | 0 | 2 | 0 | 0 | 0 | 1 | 0 |
| RUM | CLY | 2 | 0 | 0 | 0 | 0 | 0 | 0 | 0 |
| RUM | CLY | 3 | 0 | 2 | 0 | 0 | 0 | 0 | 0 |
| RUM | CLY | 4 | 0 | 0 | 0 | 0 | 0 | 0 | 0 |
| RUM | CLY | 5 | 0 | 5 | 0 | 0 | 0 | 0 | 0 |
| RUM | CLY | 6 | 0 | 1 | 0 | 0 | 0 | 0 | 0 |
| RUM | CLY | 7 | 0 | 2 | 0 | 0 | 0 | 0 | 0 |

| **Mo** | **Es** | **Rep** | **Nereid sp.1** | ***Oenone* sp.** | ***Armandia intermedia*** | ***Scoloplos (Leodomos) johnstoni*** | ***Scoloplos (Scoloplos) simplex*** | ***Aricidea* sp.** |
| --- | --- | --- | --- | --- | --- | --- | --- | --- |
| RUM | HAK | 1 | 0 | 0 | 0 | 0 | 0 | 0 |
| RUM | HAK | 2 | 0 | 0 | 0 | 1 | 0 | 0 |
| RUM | HAK | 3 | 0 | 0 | 0 | 0 | 0 | 0 |
| RUM | HAK | 4 | 0 | 0 | 0 | 1 | 0 | 0 |
| RUM | HAK | 6 | 0 | 0 | 0 | 0 | 0 | 0 |
| RUM | HAK | 7 | 0 | 0 | 0 | 0 | 0 | 0 |
| RUM | WAG | 1 | 0 | 0 | 0 | 0 | 0 | 0 |
| RUM | WAG | 2 | 0 | 0 | 0 | 0 | 0 | 1 |
| RUM | WAG | 3 | 0 | 0 | 0 | 1 | 1 | 0 |
| RUM | WAG | 4 | 0 | 0 | 0 | 0 | 0 | 0 |
| RUM | WAG | 5 | 0 | 0 | 1 | 0 | 0 | 0 |
| RUM | WAG | 6 | 0 | 0 | 0 | 0 | 0 | 1 |
| RUM | WAG | 7 | 0 | 0 | 0 | 0 | 0 | 0 |
| RUM | CLY | 1 | 0 | 0 | 0 | 0 | 0 | 0 |
| RUM | CLY | 2 | 0 | 0 | 0 | 0 | 0 | 0 |
| RUM | CLY | 3 | 0 | 0 | 0 | 0 | 0 | 0 |
| RUM | CLY | 4 | 0 | 0 | 1 | 0 | 0 | 0 |
| RUM | CLY | 5 | 0 | 0 | 0 | 0 | 0 | 1 |
| RUM | CLY | 6 | 0 | 0 | 0 | 0 | 0 | 0 |
| RUM | CLY | 7 | 0 | 0 | 0 | 0 | 0 | 0 |

| **Mo** | **Es** | **Rep** | ***Paraonis* sp.** | ***Pectinaria australis*** | ***Paralepidonotus ampulliferus*** | ***Euchone variabilis*** | ***Prionospio* *wambiri*** | ***Prionospio tatura*** |
| --- | --- | --- | --- | --- | --- | --- | --- | --- |
| RUM | HAK | 1 | 0 | 0 | 1 | 0 | 0 | 0 |
| RUM | HAK | 2 | 0 | 0 | 0 | 0 | 0 | 3 |
| RUM | HAK | 3 | 0 | 0 | 1 | 0 | 2 | 2 |
| RUM | HAK | 4 | 0 | 0 | 0 | 0 | 0 | 2 |
| RUM | HAK | 6 | 0 | 0 | 0 | 0 | 2 | 3 |
| RUM | HAK | 7 | 0 | 0 | 1 | 0 | 2 | 2 |
| RUM | WAG | 1 | 0 | 0 | 0 | 0 | 0 | 0 |
| RUM | WAG | 2 | 0 | 0 | 0 | 0 | 0 | 2 |
| RUM | WAG | 3 | 0 | 0 | 1 | 0 | 0 | 1 |
| RUM | WAG | 4 | 0 | 0 | 0 | 0 | 0 | 1 |
| RUM | WAG | 5 | 1 | 0 | 0 | 0 | 0 | 2 |
| RUM | WAG | 6 | 2 | 0 | 0 | 0 | 0 | 3 |
| RUM | WAG | 7 | 0 | 0 | 2 | 0 | 0 | 0 |
| RUM | CLY | 1 | 1 | 0 | 0 | 0 | 0 | 0 |
| RUM | CLY | 2 | 0 | 0 | 1 | 0 | 0 | 0 |
| RUM | CLY | 3 | 0 | 0 | 0 | 0 | 0 | 1 |
| RUM | CLY | 4 | 0 | 0 | 0 | 0 | 0 | 0 |
| RUM | CLY | 5 | 0 | 0 | 0 | 0 | 0 | 0 |
| RUM | CLY | 6 | 0 | 0 | 0 | 0 | 0 | 0 |
| RUM | CLY | 7 | 0 | 1 | 0 | 0 | 0 | 0 |

| **Mo** | **Es** | **Rep** | ***Paraprionospio* sp.** | ***Prionospio yuriel*** | ***Pseudopolydora* sp*.*** | ***Carraziella* sp.** | ***Polydora* sp.** | **Exogene sp.1** | **Exogene sp. 2** |
| --- | --- | --- | --- | --- | --- | --- | --- | --- | --- |
| RUM | HAK | 1 | 0 | 1 | 4 | 0 | 0 | 0 | 0 |
| RUM | HAK | 2 | 1 | 0 | 1 | 0 | 0 | 0 | 0 |
| RUM | HAK | 3 | 1 | 0 | 0 | 0 | 0 | 0 | 0 |
| RUM | HAK | 4 | 2 | 0 | 0 | 0 | 0 | 0 | 0 |
| RUM | HAK | 6 | 2 | 0 | 0 | 0 | 0 | 0 | 0 |
| RUM | HAK | 7 | 0 | 0 | 6 | 0 | 0 | 0 | 0 |
| RUM | WAG | 1 | 1 | 0 | 0 | 0 | 0 | 0 | 0 |
| RUM | WAG | 2 | 1 | 0 | 0 | 0 | 0 | 2 | 0 |
| RUM | WAG | 3 | 1 | 0 | 4 | 0 | 0 | 1 | 0 |
| RUM | WAG | 4 | 1 | 0 | 1 | 0 | 0 | 1 | 1 |
| RUM | WAG | 5 | 1 | 0 | 2 | 0 | 0 | 0 | 0 |
| RUM | WAG | 6 | 0 | 0 | 0 | 0 | 0 | 2 | 0 |
| RUM | WAG | 7 | 2 | 0 | 1 | 0 | 0 | 0 | 1 |
| RUM | CLY | 1 | 1 | 0 | 0 | 0 | 0 | 1 | 0 |
| RUM | CLY | 2 | 0 | 0 | 0 | 0 | 0 | 0 | 0 |
| RUM | CLY | 3 | 1 | 0 | 2 | 0 | 0 | 0 | 0 |
| RUM | CLY | 4 | 1 | 0 | 0 | 0 | 0 | 0 | 0 |
| RUM | CLY | 5 | 0 | 0 | 1 | 0 | 1 | 1 | 0 |
| RUM | CLY | 6 | 1 | 0 | 2 | 0 | 0 | 1 | 0 |
| RUM | CLY | 7 | 4 | 0 | 3 | 0 | 1 | 2 | 0 |

| **Mo** | **Es** | **Rep** | **Exogene sp.3** | ***Astreptosyllis similiseta*** | **Syllinae sp.1** | **Syllinae sp.2** | ***Syllis* cf. *chrysillus*** | **Syllid/Phyllodocidae** | ***Lysilla* sp.** |
| --- | --- | --- | --- | --- | --- | --- | --- | --- | --- |
| RUM | HAK | 1 | 0 | 0 | 0 | 0 | 0 | 0 | 0 |
| RUM | HAK | 2 | 1 | 0 | 0 | 0 | 0 | 0 | 0 |
| RUM | HAK | 3 | 0 | 0 | 0 | 0 | 0 | 0 | 0 |
| RUM | HAK | 4 | 1 | 1 | 0 | 0 | 0 | 0 | 0 |
| RUM | HAK | 6 | 0 | 0 | 0 | 0 | 0 | 0 | 0 |
| RUM | HAK | 7 | 0 | 0 | 0 | 0 | 1 | 0 | 0 |
| RUM | WAG | 1 | 0 | 0 | 0 | 0 | 0 | 0 | 0 |
| RUM | WAG | 2 | 0 | 2 | 0 | 0 | 0 | 0 | 0 |
| RUM | WAG | 3 | 0 | 0 | 1 | 0 | 0 | 0 | 0 |
| RUM | WAG | 4 | 0 | 1 | 0 | 0 | 0 | 0 | 0 |
| RUM | WAG | 5 | 0 | 1 | 0 | 0 | 0 | 0 | 0 |
| RUM | WAG | 6 | 0 | 0 | 0 | 0 | 0 | 0 | 0 |
| RUM | WAG | 7 | 0 | 1 | 0 | 0 | 0 | 0 | 0 |
| RUM | CLY | 1 | 0 | 0 | 0 | 0 | 0 | 0 | 0 |
| RUM | CLY | 2 | 0 | 0 | 0 | 0 | 0 | 0 | 0 |
| RUM | CLY | 3 | 0 | 0 | 0 | 0 | 0 | 0 | 0 |
| RUM | CLY | 4 | 0 | 0 | 0 | 0 | 0 | 0 | 0 |
| RUM | CLY | 5 | 0 | 0 | 0 | 0 | 0 | 0 | 0 |
| RUM | CLY | 6 | 0 | 0 | 0 | 0 | 0 | 0 | 0 |
| RUM | CLY | 7 | 0 | 0 | 0 | 0 | 0 | 0 | 0 |

| **Mo** | **Es** | **Rep** | ***Ameania* sp.** | **Oligochaete** | **Nematode** | **Nemertean** | **Sipuncula** | **Amphipoda** | **Copepoda** | **Decapoda** | **Isopoda** | **Tanaidacea** | **Cumacea** |
| --- | --- | --- | --- | --- | --- | --- | --- | --- | --- | --- | --- | --- | --- |
| RUM | HAK | 1 | 0 | 0 | 0 | 0 | 0 | 4 | 0 | 0 | 0 | 0 | 0 |
| RUM | HAK | 2 | 0 | 0 | 0 | 1 | 0 | 3 | 0 | 0 | 0 | 1 | 1 |
| RUM | HAK | 3 | 0 | 0 | 0 | 0 | 0 | 4 | 1 | 2 | 0 | 1 | 0 |
| RUM | HAK | 4 | 0 | 0 | 0 | 0 | 0 | 6 | 0 | 0 | 0 | 2 | 0 |
| RUM | HAK | 6 | 0 | 0 | 0 | 1 | 0 | 10 | 0 | 4 | 0 | 0 | 0 |
| RUM | HAK | 7 | 0 | 0 | 0 | 1 | 0 | 7 | 0 | 0 | 0 | 0 | 0 |
| RUM | WAG | 1 | 0 | 0 | 0 | 0 | 0 | 6 | 0 | 1 | 0 | 0 | 0 |
| RUM | WAG | 2 | 0 | 0 | 0 | 0 | 0 | 8 | 0 | 0 | 0 | 1 | 0 |
| RUM | WAG | 3 | 0 | 0 | 0 | 0 | 0 | 3 | 1 | 0 | 0 | 0 | 0 |
| RUM | WAG | 4 | 0 | 0 | 0 | 1 | 0 | 10 | 1 | 0 | 0 | 1 | 0 |
| RUM | WAG | 5 | 0 | 0 | 0 | 0 | 0 | 2 | 0 | 0 | 0 | 0 | 0 |
| RUM | WAG | 6 | 0 | 0 | 1 | 0 | 0 | 6 | 0 | 0 | 0 | 3 | 0 |
| RUM | WAG | 7 | 0 | 0 | 0 | 1 | 0 | 15 | 0 | 0 | 0 | 1 | 0 |
| RUM | CLY | 1 | 0 | 0 | 0 | 0 | 0 | 2 | 0 | 0 | 0 | 1 | 0 |
| RUM | CLY | 2 | 0 | 0 | 0 | 0 | 0 | 0 | 0 | 0 | 0 | 0 | 0 |
| RUM | CLY | 3 | 0 | 0 | 0 | 0 | 0 | 1 | 0 | 0 | 0 | 0 | 0 |
| RUM | CLY | 4 | 0 | 0 | 0 | 2 | 0 | 0 | 0 | 0 | 0 | 0 | 0 |
| RUM | CLY | 5 | 0 | 0 | 0 | 0 | 0 | 0 | 0 | 0 | 0 | 0 | 0 |
| RUM | CLY | 6 | 0 | 0 | 0 | 0 | 0 | 2 | 0 | 0 | 1 | 1 | 0 |
| RUM | CLY | 7 | 0 | 0 | 0 | 0 | 0 | 1 | 0 | 0 | 0 | 0 | 0 |

| **Mo** | **Es** | **Rep** | **Gastropoda** | **Juveniles** |
| --- | --- | --- | --- | --- |
| RUM | HAK | 1 | 0 | 0 |
| RUM | HAK | 2 | 0 | 0 |
| RUM | HAK | 3 | 1 | 0 |
| RUM | HAK | 4 | 0 | 0 |
| RUM | HAK | 6 | 0 | 1 |
| RUM | HAK | 7 | 0 | 0 |
| RUM | WAG | 1 | 0 | 0 |
| RUM | WAG | 2 | 0 | 0 |
| RUM | WAG | 3 | 0 | 1 |
| RUM | WAG | 4 | 0 | 1 |
| RUM | WAG | 5 | 0 | 0 |
| RUM | WAG | 6 | 0 | 1 |
| RUM | WAG | 7 | 0 | 0 |
| RUM | CLY | 1 | 0 | 0 |
| RUM | CLY | 2 | 0 | 0 |
| RUM | CLY | 3 | 0 | 0 |
| RUM | CLY | 4 | 0 | 0 |
| RUM | CLY | 5 | 0 | 0 |
| RUM | CLY | 6 | 0 | 0 |
| RUM | CLY | 7 | 0 | 0 |
