# Supplementary material for: Polychaete Richness and Abundance Enhanced in Anthropogenically Modified Estuaries Despite High Concentrations of Toxic Contaminants
Source: PLoS One. 2013 Sep 30;8(9):e77018. doi: 10.1371/journal.pone.0077018 (PMC3786951; doi:10.1371/journal.pone.0077018)
Supplement: Table S4 — Permutational ANOVA results for sediment quality measures in benthic sediment surveyed from seven NSW estuaries. (DOCX) [file pone.0077018.s008.docx]

**Table S4.** Permutational ANOVA results for sediment quality measures in benthic sediment surveyed from seven NSW estuaries. Factors include Modification category (Mo; heavily modified or relatively unmodified) and Estuary (Es; nested in Modification category). Sites were the replicates. Values in bold are significant.

| **Source** | **df** | **SS** | **MS** | **Psuedo-F** | **P(perm)** |  | **SS** | **MS** | **Psuedo-F** | **P(perm)** |
| --- | --- | --- | --- | --- | --- | --- | --- | --- | --- | --- |
|  |  | **Chromium** | |  |  |  | **Copper** |  |  |  |
| Modification category | 1 | 24.03 | 24.03 | 25.76 | **0.017** |  | 24.67 | 24.67 | 12.98 | **0.013** |
| Estuary (Mo) | 5 | 4.66 | 0.93 | 2.14 | 0.078 |  | 9.50 | 1.90 | 6.28 | **0.000** |
| Res | 39 | 17.02 | 0.44 |  |  |  | 11.81 | 0.30 |  |  |
|  |  | **Nickel** |  |  |  |  | **Lead** | |  |  |
| Modification category | 1 | 20.84 | 20.84 | 15.17 | 0.051 |  | 22.29 | 22.29 | 11.29 | **0.024** |
| Estuary (Mo) | 5 | 6.86 | 1.37 | 3.00 | **0.021** |  | 9.86 | 1.97 | 6.11 | **0.000** |
| Res | 39 | 17.83 | 0.46 |  |  |  | 12.58 | 0.32 |  |  |
|  |  | **Zinc** |  |  |  |  | **Total PAHs** | |  |  |
| Modification category | 1 | 23.15 | 23.15 | 16.95 | **0.018** |  | 12.23 | 12.23 | 3.92 | **0.007** |
| Estuary (Mo) | 5 | 6.82 | 1.36 | 3.41 | **0.011** |  | 15.59 | 3.12 | 6.57 | **0.000** |
| Res | 39 | 15.62 | 0.40 |  |  |  | 18.51 | 0.47 |  |  |
|  |  | **Sediment Chl-a** | |  |  |  | **Total organic carbon** | | |  |
| Modification category | 1 | 16.54 | 16.54 | 21.00 | **0.009** |  | 11.05 | 11.05 | 3.32 | 0.100 |
| Estuary (Mo) | 5 | 3.94 | 0.79 | 1.29 | 0.283 |  | 16.60 | 3.32 | 6.99 | **0.000** |
| Res | 39 | 23.85 | 0.61 |  |  |  | 18.53 | 0.48 |  |  |
|  |  | **Porewater ammonia** | | |  |  | **Percent fines** | | | |
| Modification category | 1 | 12.36 | 12.36 | 19.86 | **0.009** |  | 15.92 | 15.92 | 16.86 | 0.053 |
| Estuary (Mo) | 5 | 3.11 | 0.62 | 0.80 | 0.547 |  | 4.72 | 0.94 | 1.53 | 0.200 |
| Res | 39 | 30.17 | 0.77 |  |  |  | 24.00 | 0.62 |  |  |
