# Supplementary material for: Polychaete Richness and Abundance Enhanced in Anthropogenically Modified Estuaries Despite High Concentrations of Toxic Contaminants
Source: PLoS One. 2013 Sep 30;8(9):e77018. doi: 10.1371/journal.pone.0077018 (PMC3786951; doi:10.1371/journal.pone.0077018)
Supplement: Table S5 — Permutational ANOVA results for water quality variables surveyed from seven NSW estuaries. (DOCX) [file pone.0077018.s009.docx]

**Table S5.** Permutational ANOVA results for water quality variables surveyed from seven NSW estuaries. Factors include Modification category (Mo; heavily modified or relatively unmodified) and Estuary (Es; nested in Modification category). Sites were the replicates. Values in bold are significant.

| **Source** | **df** | **SS** | **MS** | **Psuedo-F** | **P(perm)** |  | **SS** | **MS** | **Pseudo-F** | **P(perm)** |
| --- | --- | --- | --- | --- | --- | --- | --- | --- | --- | --- |
|  |  | **Salinity** |  |  |  |  | **Temperature** | |  |  |
| Modification category | 1 | 1.95 | 1.95 | 0.29 | 0.610 |  | 0.11 | 0.11 | 0.14 | 0.743 |
| Estuary (Mo) | 5 | 33.74 | 6.75 | 27.02 | **0.000** |  | 4.20 | 0.84 | 0.80 | 0.664 |
| Res | 39 | 9.74 | 0.25 |  |  |  | 40.71 | 1.04 |  |  |
|  |  | **pH** |  |  |  |  | **Turbidity** | |  |  |
| Modification category | 1 | 2.16 | 2.16 | 0.83 | 0.380 |  | 11.22 | 11.22 | 3.94 | **0.012** |
| Estuary (Mo) | 5 | 13.08 | 2.62 | 3.39 | **0.012** |  | 14.22 | 2.84 | 5.96 | **0.000** |
| Res | 39 | 30.08 | 0.77 |  |  |  | 18.60 | 0.48 |  |  |
|  |  | **Dissolved oxygen** | | | |  | **Water Chl-a** | | | |
| Modification category | 1 | 0.21 | 0.21 | 0.09 | 0.813 |  | 0.16 | 0.16 | 0.07 | 0.743 |
| Estuary (Mo) | 5 | 10.98 | 2.20 | 2.53 | **0.040** |  | 11.29 | 2.26 | 2.63 | **0.035** |
| Res | 39 | 33.87 | 0.87 |  |  |  | 33.55 | 0.86 |  |  |
