# Supplementary material for: Polychaete Richness and Abundance Enhanced in Anthropogenically Modified Estuaries Despite High Concentrations of Toxic Contaminants
Source: PLoS One. 2013 Sep 30;8(9):e77018. doi: 10.1371/journal.pone.0077018 (PMC3786951; doi:10.1371/journal.pone.0077018)
Supplement: Table S6 — Permutational ANOVA results for metal contaminants and sediment quality variables measured at the start and end in sediment field experiments deployed in the Clyde estuary. (DOCX) [file pone.0077018.s010.docx]

**Table S6.** Permutational ANOVA results for metal contaminants and sediment quality variables measured at the start and end in sediment field experiments deployed in the Clyde estuary. Factors include Modification category (Mo; heavily modified or relatively unmodified) and Estuary (Es; nested in Modification category). Estuary or BRCs were the replicates. Values in bold are significant.

| **Source** | **df** | **SS** | **MS** | **Pseudo-F** | **P(perm)** |  | **SS** | **MS** | **Pseudo-F** | **P(perm)** |
| --- | --- | --- | --- | --- | --- | --- | --- | --- | --- | --- |
|  |  | **Copper (start)** | |  |  |  | **Lead (start)** | |  |  |
| Modification category | 1 | 26.85 | 26.85 | 75.91 | **0.000** |  | 25.08 | 25.08 | 62.99 | **0.000** |
| Res | 40 | 14.15 | 0.35 |  |  |  | 15.92 | 0.40 |  |  |
|  |  |  |  |  |  |  |  |  |  |  |
|  |  | **Zinc (start)** | |  |  |  | **Percent fines** | |  |  |
| Modification category | 1 | 31.44 | 31.44 | 131.56 | **0.000** |  | 21.56 | 21.56 | 44.35 | **0.000** |
| Res | 40 | 9.56 | 0.24 |  |  |  | 19.44 | 0.49 |  |  |
|  |  |  |  |  |  |  |  |  |  |  |
|  |  | **Copper (end)** | |  |  |  | **Lead (end)** | |  |  |
| Modification category | 1 | 12.69 | 12.69 | 8.25 | **0.044** |  | 12.00 | 12.00 | 8.84 | **0.040** |
| Es (Mo) | 4 | 6.15 | 1.54 | 2.50 | 0.057 |  | 5.43 | 1.36 | 2.07 | 0.104 |
| Res | 36 | 22.15 | 0.62 |  |  |  | 23.57 | 0.65 |  |  |
|  |  |  |  |  |  |  |  |  |  |  |
|  |  | **Zinc (end)** | |  |  |  | **Percent fines** | |  |  |
| Modification category | 1 | 13.81 | 13.81 | 10.10 | **0.031** |  | 9.53 | 9.53 | 5.55 | 0.070 |
| Es (Mo) | 4 | 5.47 | 1.37 | 2.27 | 0.080 |  | 6.87 | 1.72 | 2.51 | 0.059 |
| Res | 36 | 21.72 | 0.60 |  |  |  | 24.60 | 0.68 |  |  |
|  |  |  |  |  |  |  |  |  |  |  |
